# Synergistic Ruthenium-Doped Amorphous IrO_*x*_ Matrix for Robust Oxygen Evolution

**DOI:** 10.1021/acsami.5c13101

**Published:** 2025-09-19

**Authors:** Jiandong Hu, Yangfan Liu, Yanlin Jia, Ziye Li, Haowei Yang, Yang Wang, Wenhui Luo, Zhi Liang Zhao, Yejun Li, Yong Pang, Qi Wang

**Affiliations:** † School of Materials Science and Engineering, 12570 Central South University, Changsha 410083, Hunan, P. R. China; ‡ Foshan Xianhu Laboratory, National energy key laboratory for new hydrogen-ammonia energy technologies, Foshan 528200, Guangdong, P. R. China; § Department of Materials Science and Engineering, 53025 City University of Hong Kong, Hong Kong 999077, P. R. China

**Keywords:** electrocatalyst, Ru-doped IrO_*x*_ nanosheets, acidic oxygen evolution reaction, electronic structure engineering, AEM mechanism

## Abstract

Iridium oxides (IrO_*x*_) are benchmark catalysts for the acidic oxygen evolution reaction, but their performance is often constrained by a trade-off between catalytic activity and long-term stability. Herein, we utilize an amorphous IrO_*x*_ matrix as a robust scaffold for synergistic ruthenium (Ru) doping, a strategy designed to enhance catalytic activity while maintaining an exceptional stability. A simple nitrate-assisted synthesis produces ultrathin Ru-doped amorphous IrO_*x*_ nanosheets (2.36 nm thick) with a significantly enhanced specific surface area. Combined spectroscopic analysis and density functional theory calculations reveal that atomically dispersed Ru dopants induce charge transfer to adjacent Ir sites, which optimizes the Ir d-band electronic structure. This electronic modulation not only lowers the energy barrier for the rate-determining *O to *OOH transformation but also critically ensures the reaction proceeds via the stable adsorbate evolution mechanism while suppressing the degradative lattice oxygen mechanism. Benefiting from the above advantages, the optimized Ru_0.0738_–IrO_*x*_ catalyst exhibits excellent catalytic activity, achieving 10 mA cm^–2^ at a low overpotential of 225 mV with outstanding stability for over 100 h, far surpassing commercial IrO_2_ and RuO_2_. This study highlights a synergistic doping strategy within an amorphous matrix to overcome the intrinsic performance limitations of iridium-based oxides for robust oxygen evolution.

## Graphical abstract

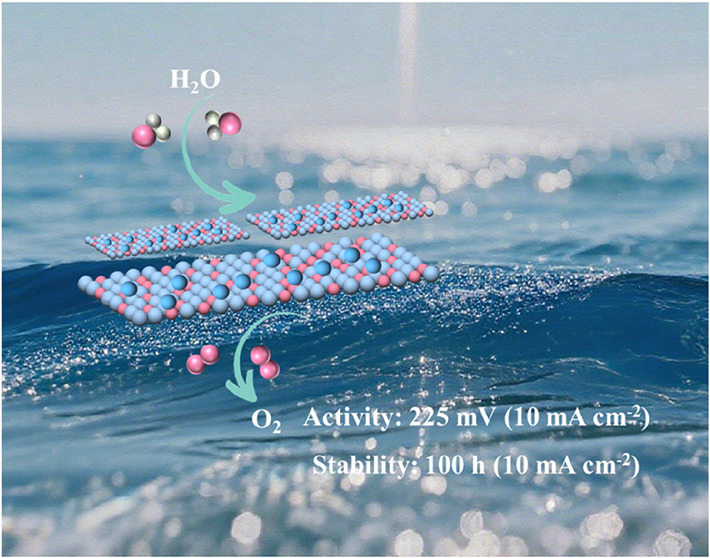

## Introduction

Proton exchange membrane water electrolyzers (PEMWEs) represent a premier technology for green hydrogen production, prized for their high current density, rapid response, and compact design, which give them a distinct advantage over traditional alkaline electrolyzers.^1–4^ However, the overall efficiency and economic viability of this promising technology are fundamentally limited by the sluggish kinetics of the anodic oxygen evolution reaction (OER), which must operate within the PEMWEs’ highly corrosive acidic environment.^5–9^ The development of electrocatalysts that are simultaneously cost-effective, highly active, and exceptionally stable under these harsh conditions therefore remains the critical bottleneck impeding the large-scale application of PEMWEs. While crystalline iridium oxide (IrO_2_) is the established benchmark for acidic OER, its performance is inherently constrained by a well-known trade-off between catalytic activity and operational stability.^10–13^ Therefore, exploring innovative strategies to transcend this limitation and fully unlock the catalytic potential of iridium-based materials is a central goal in the field.^14–16^ Among the various approaches, engineering nanostructured amorphous materials has emerged as a particularly effective strategy.^17–19^ Compared with their crystalline counterparts, amorphous IrO_*x*_ exhibits a higher ratio of exposed atoms and unsaturated coordination sites, which enhances its intrinsic activity. Furthermore, the isotropy and distinctive internal nature of amorphous IrO_*x*_ can confer superior corrosion resistance, a vital attribute for durable OER catalysis.^20–23^ Unfortunately, the absence of straightforward protocols for the synthesis process, elevated temperatures, and pressure may result in the recrystallization of amorphous IrO_*x*_.^24–26^

Besides, the catalyst morphology, as a crucial element of the surface, has a direct impact on the electrochemical surface area, the number of exposed active sites, and the diffusive mass transfer of the electrolyte.^25,27^ Two-dimensional (2D) nanosheets offer numerous electrochemically active sites and enhanced surface functionality due to the high proportion of surface atoms. This leads to increased surface area, improved mechanical flexibility, faster interfacial charge transfer, and more facile electrochemical reactions.^22,28–30^ Beyond structural and morphological control, tuning the electronic structure of the active sites via elemental doping offers a powerful lever to enhance intrinsic catalysis.^31,32^ The incorporation of heteroatoms can modulate the d-band center of Ir, optimize the adsorption energies of OER intermediates, and create synergistic effects that boost performance.^33,34^ Thus, selecting an optimal element doping strategy for electronic structure modification represents a straightforward and effective approach.^34–36^ For instance, doping IrO_2_ with neodymium (Nd) was shown to optimize the Ir electronic structure but also introduced oxygen vacancies that activated the degradative lattice oxygen mechanism (LOM), thereby compromising stability.^37,17,38–40^ Consequently, optimizing the electronic structure of Ir-based catalysts via elemental doping and preventing LOM represents a challenge.

In this study, we address this challenge by developing a synergistic catalyst composed of ruthenium (Ru) dopants within an amorphous IrO_*x*_ nanosheet matrix. The results show that amorphous Ru_0.0738_-IrO_*x*_ has a nanosheet thickness of 2.36 nm and exhibits a remarkable specific surface area. XAS indicates that the moderate amount of Ru doping on the one hand effectively induces the charge transfer from Ru to Ir and optimizes the electron density distribution around Ir. On the other hand, it reduces the covalency of Ir–O bonds, which enables the IrO_*x*_ matrix to maintain the AEM in the OER process and effectively avoids the activation of the LOM. Corresponding density functional theory (DFT) calculations show that Ru doping effectively optimizes the projected density of states (PDOS) in the center of the IrO_*x*_ d-band, which in turn drastically reduces the energy barrier required for OER to occur. As a result, the representative catalyst Ru_0.0738_-IrO_*x*_ exhibits excellent catalytic activity and stability, which are significantly better than those of commercial IrO_2_ and commercial RuO_2_. The present work clarifies Ru-doped amorphous IrO_*x*_ with an ultrathin nanosheet structure as a promising catalyst, opening a new route for the development of high-performance catalysts.

## Results and Discussion

### Materials Synthesis and Characterization

The synthesis of Ru-doped amorphous IrO_*x*_ nanosheets, designated as Ru_*y*_-IrO_*x*_ (where *y* represents the nominal Ru:Ir molar ratio), was conducted following the method illustrated in Figure 1a (see Methods section in Supporting Information for details). Actual Ru doping levels, determined by inductively coupled plasma-mass spectrometry (ICP-MS) and presented in Table S1, were largely consistent with the nominal doping levels, with a slight deviation likely due to Ru losses during the synthesis process. As illustrated in Figure 1b, the XRD patterns for both pristine IrO_*x*_ and Ru_*y*_-IrO_*x*_ samples with Ru:Ir molar ratios up to *y* = 0.0738 predominantly displayed broad, diffuse humps. These features are characteristic of an amorphous structure, signifying the absence of long-range crystallographic order. Notably, when the nominal Ru:Ir molar ratio was increased beyond a critical threshold of approximately 0.0983 (as observed in samples Ru_0.0983_-IrO_*x*_ and Ru_0.148_-IrO_*x*_), the XRD patterns (Figure S1) revealed the emergence of distinct, sharp Bragg peaks. These peaks are unequivocally attributable to a crystalline RuO_2_ phase, indicating that Ru doping beyond this solubility limit within the amorphous IrO_*x*_ matrix leads to phase segregation.

**Figure 1:**
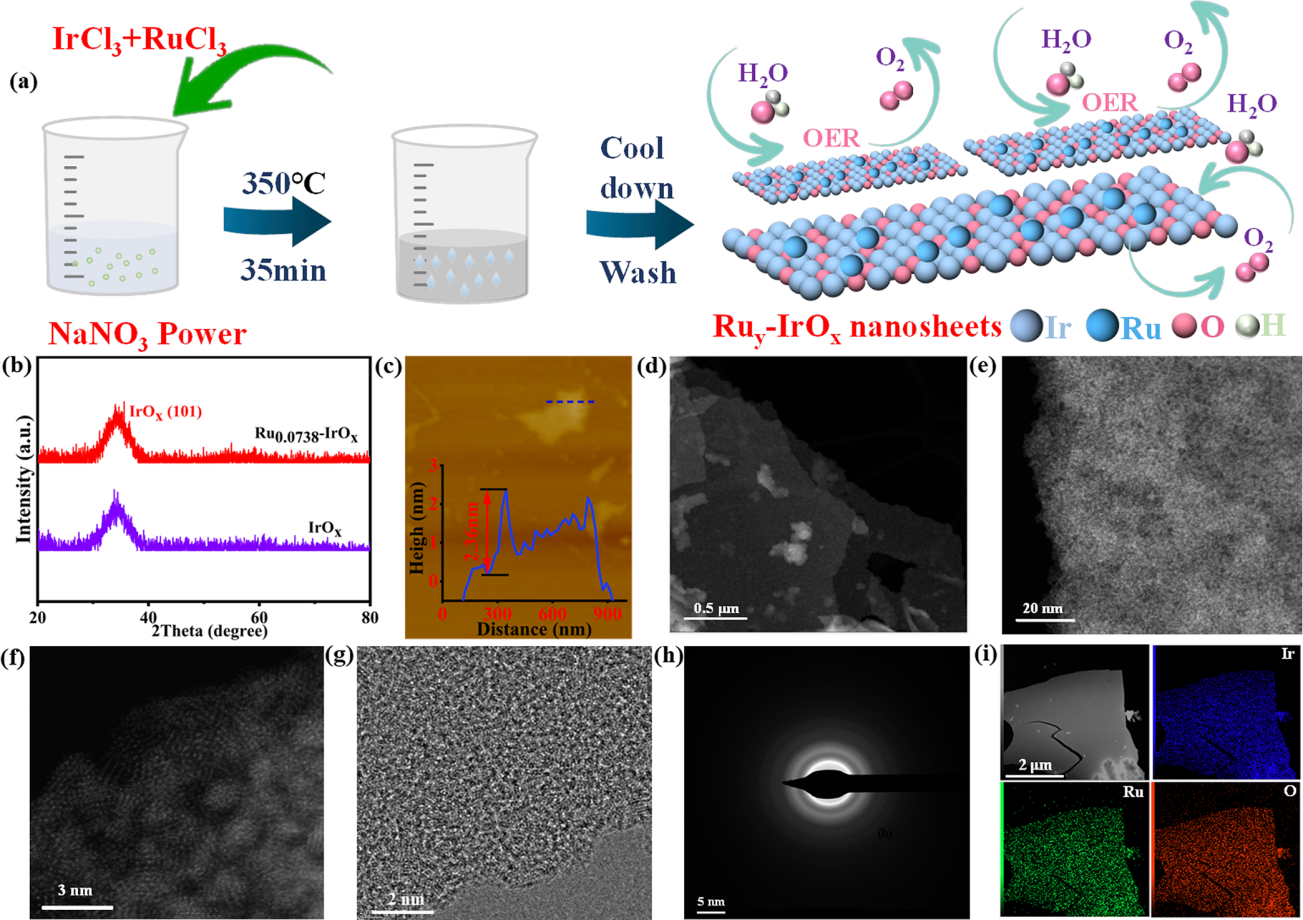
(a) Schematic representation of the synthesis of Ru_*y*_-IrO_*x*_. (b) XRD patterns of IrO_*x*_ and Ru_0.0738_-IrO_*x*_. (c) AFM image of Ru_0.0738_-IrO_*x*_. (d) Low-magnification STEM image of Ru_0.0738_-IrO_*x*_. (e, f) High-resolution STEM images of Ru_0.0738_-IrO_*x*_. (g) High-resolution TEM image of Ru_0.0738_-IrO_*x*_. (h). SAED image of Ru_0.0738_-IrO_*x*_. (i) EDS mapping of Ru_0.0738_-IrO_*x*_ for Ir, Ru, and O and the combined image.

The morphology and atomic structure of the representative Ru_0.0738_-IrO_*x*_ catalyst were further investigated by using scanning transmission electron microscopy (STEM) and atomic force microscopy (AFM). STEM images (Figures 1d and S2) confirm the nanosheet morphology of the Ru_0.0738_-IrO_*x*_. AFM analysis (Figures 1c and S3) showed that the thickness of Ru_0.0738_-IrO_*x*_ was about 2.36 nm, which is significantly thinner than the undoped IrO_*x*_ (7.32 nm). Such ultrathin nanosheet architectures are anticipated to provide a high surface-to-volume ratio, abundant mass diffusion channels, efficient charge transport pathways, and highly exposed active planes, all of which are beneficial for the electrocatalytic reaction.^28^ This was corroborated by Brunauer–Emmett–Teller (BET) surface area analysis (Figure S4), where Ru_0.0738_-IrO_*x*_ exhibited a high specific surface area of 203.9 m^2^/g, significantly larger than that of undoped IrO_*x*_ (105.2919 m^2^/g), commercial RuO_2_ (26.92 m^2^/g) and commercial IrO_2_ (14.19 m^2^/g). This increased surface area suggests a greater availability of accessible active sites for the OER. The atomic and nanoscale structures of the representative Ru_0.0738_-IrO_*x*_ were further elucidated through detailed electron microscopy. Atomic-resolution STEM images (Figure 1e,1f) directly visualized the atomic arrangement, revealing a long-range disordered structure consistent with an amorphous phase. This lack of crystallinity was further corroborated by a high-resolution TEM image (HRTEM). The HRTEM image (Figure 1g) of a typical nanosheet displayed no discernible lattice fringes, which would otherwise indicate periodic atomic arrangements. Consistently, the corresponding selected area electron diffraction (SAED) pattern (Figure 1h) exhibited a distinct diffuse diffraction halo, a characteristic fingerprint of amorphous materials, rather than discrete diffraction spots expected from a crystalline structure. The corresponding energy-dispersive spectroscopy (EDS) mapping (Figure 1i) demonstrates that the elements Ir, Ru, and O are distributed uniformly in Ru_0.0738_-IrO_*x*_ and the presence of the element Ru was also confirmed by X-ray photoelectron spectroscopy (XPS) analysis (Figure S5a). The above analyses demonstrate the successful synthesis of amorphous Ru_0.0738_-IrO_*x*_ nanosheets.

### Electronic Structure of Ru_0.0738_-IrO_*x*_

XPS was employed to analyze the electronic structure and valence states of the Ru_0.0738_-IrO_*x*_ catalyst, comparing it to those of pure IrO_*x*_, commercial IrO_2_, and commercial RuO_2_. Full XPS spectra confirmed the successful incorporation of Ru within the IrO_*x*_ matrix (Figure S5a). The high-resolution Ir 4f spectrum of Ru_0.0738_-IrO_*x*_ (Figure 2a) exhibits peaks at 61.65, 62.2, and 63.74 eV, corresponding to Ir^3+^, Ir^4+^, and Ir satellite peaks for Ir 4f_7/2_, respectively. Peaks at 64.75, 65.3, and 67.01 eV are assigned to Ir^3+^, Ir^4+^, and Ir satellite peaks of Ir 4f_5/2_, respectively.^41^ Critically, a distinct negative shift in the Ir 4f peak positions was observed for Ru_0.0738_-IrO_*x*_ compared to those of undoped IrO_*x*_ and C-IrO_2_. This trend was consistent across different Ru doping levels (Figure S5b). These observations suggest charge transfer from Ru to Ir, leading to charge enrichment around Ir and a lower Ir valence state.^32^ The Ru 3d core-level spectrum, which overlaps with the C 1s signal, was carefully analyzed (Figure S5c). After the adventitious carbon signals (C–C at ∼284.6 eV and C=O at ∼288.2 eV) were accounted for, peaks corresponding to Ru species were identified. The peaks at approximately 281.35 eV (Ru 3d_5/2_) and 285.36 eV (Ru 3d_3/2_) are attributed to Ru^0^ species, while those at 282.36 eV (Ru 3d_5/2_) and 286.36 eV (Ru 3d_3/2_) are assigned to Ru^>4+^ species.^42^ These Ru peaks exhibit a positive shift compared with those of commercial RuO_2_. Further supporting this, the Ru 3p spectrum (Figure 2b) also showed peaks (Ru 3p_3/2_ at ∼462.6 and ∼465.0 eV; Ru 3p_1/2_ at ∼484.5 and ∼486.7 eV) assigned to Ru^0^ and Ru^>4+^, respectively.^43^ These Ru 3p features also show a clear positive shift compared to commercial RuO_2_. The consistent positive shift in both Ru 3d and Ru 3p binding energies across various Ru doping concentrations (Figure S5c,d) indicates that the Ru in Ru_0.0738_-IrO_*x*_ is in a higher average oxidation state than in C-RuO_2_, likely due to electron donation to Ir.^44^ Analysis of the O 1s spectra (Figures 2c and S5e) reveals different oxygen species. In Ru_0.0738_-IrO_*x*_, the peak at 530.39 eV is attributed to the Ir–O lattice bond, the peak at 532.96 eV to surface C=O bonds, and the peak at 531.59 eV to the OH species on the catalyst surface.^24^

**Figure 2:**
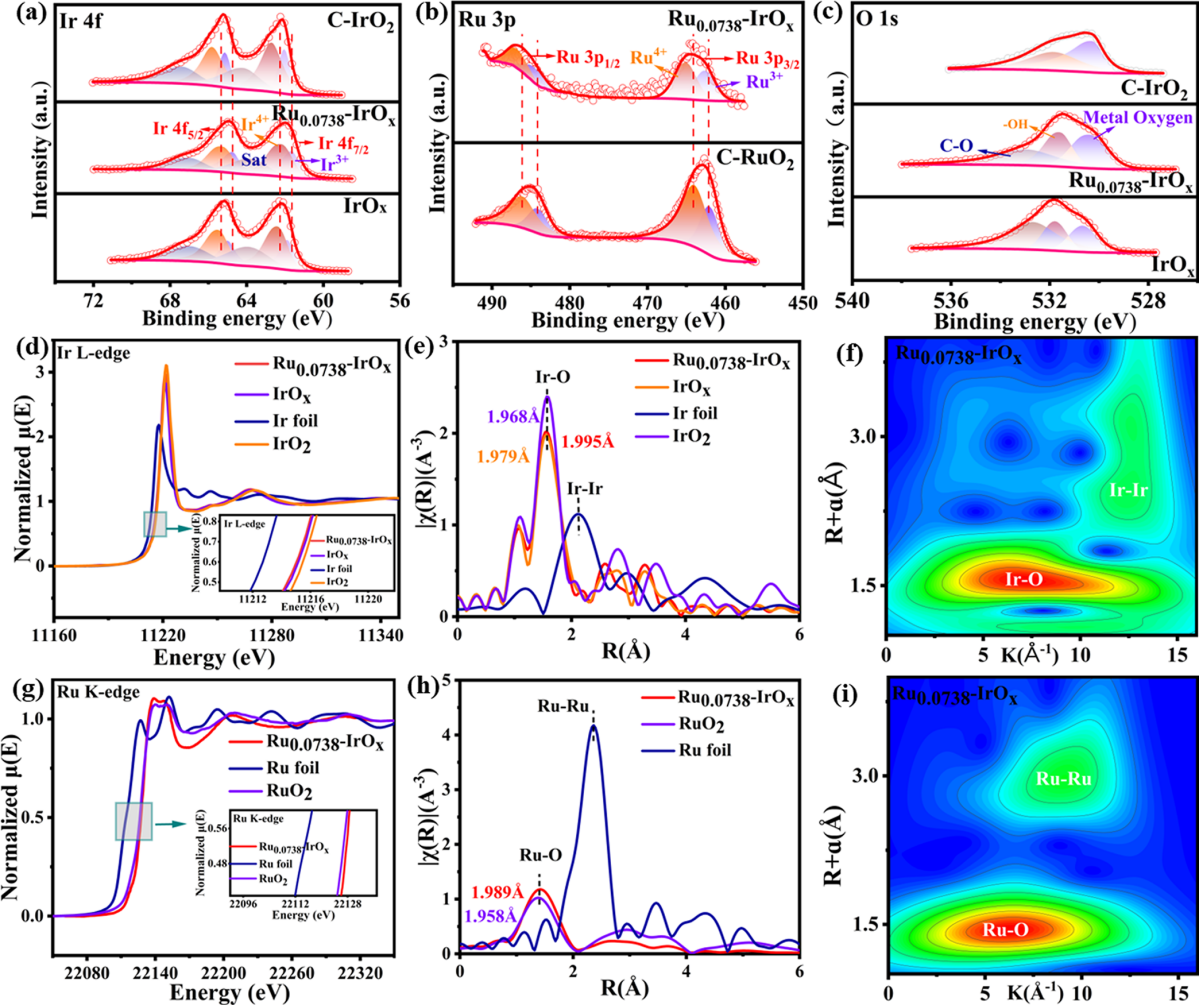
(a) High-resolution XPS spectrum of Ir 4f of Ru_0.0738_-IrO_*x*_, IrO_*x*_, and C-IrO_2_. (b) High-resolution XPS of Ru 3p of Ru_0.0738_-IrO_*x*_ and C-RuO_2_. (c) High-resolution XPS of O 1s of Ru_0.0738_-IrO_*x*_, IrO_*x*_, and C-IrO_2_. (d) Normalized XANES spectra of Ru_0.0738_-IrO_*x*_, IrO_*x*_, C-IrO_2_, and Ir foil at the Ir L-edge, inset: enlarged XANES spectra. (e) *R*-space of the EXAFS spectra at the Ir L-edge for Ru_0.0738_-IrO_*x*_, IrO_*x*_, C-IrO_2_, and Ir foil. (f) WT analysis of the *k* ^2^-weight Ir L-edge EXAFS signal of Ru_0.0738_-IrO_*x*_. (g) Normalized XANES spectra of Ru_0.0738_-IrO_*x*_, RuO_2_, and Ru foil at the Ru K-edge, inset: enlarged XANES spectra. (h) *R*-space of the EXAFS spectra at the Ru K-edge for Ru_0.0738_-IrO_*x*_, RuO_2_, and Ru foil. (i) WT analysis of the *k* ^2^-weight Ru K-edge EXAFS signal of Ru_0.0738_-IrO_*x*_.

X-ray absorption spectroscopy (XAS) was employed to analyze the valence states and coordination environments surrounding the Ir and Ru atoms in Ru_0.0738_-IrO_*x*_, comparing it to pure IrO_*x*_, commercial IrO_2_, Ir foil, commercial RuO_2_, and Ru foil. The Ir L_3_-edge X-ray absorption near-edge structure (XANES) spectra demonstrated that the absorption edges of Ru_0.0738_-IrO_*x*_ are situated between those of undoped IrO_*x*_ and IrO_2_, but are notably shifted to a slightly lower energy compared to pristine IrO_*x*_ (Figure 2d). This suggests that the valence state of Ru_0.0738_-IrO_*x*_ is marginally lower than that of IrO_*x*_ and IrO_2_, which is corroborated by the XPS results. Analysis of the Fourier-transformed (FT) extended X-ray absorption fine structure (EXAFS) spectra (Figures 2e and S6, Table S2) revealed that the main Ir–O coordination path in Ru_0.0738_-IrO_*x*_ (at 1.995 Å) is longer than that in pristine IrO_*x*_ (1.979 Å) and C-IrO_2_ (1.968 Å). This elongation of the Ir–O bond upon Ru doping suggests a decrease in the covalency of the Ir–O interaction.^45^ Furthermore, the reduced intensities of the second and third Ir–Ir coordination shells in Ru_0.0738_-IrO_*x*_ compared to crystalline references signify a diminished long-range order, consistent with its amorphous nature.^23^ By performing the wavelet transform (WT) on the Ir K-edge spectra (Figures 2f and S7), it was found that the maximum intensity of the WT contour plot of Ru_0.0738_-IrO_*x*_ is located near 6.5 Å in comparison with IrO_*x*_ and IrO_2_. This is attributed to the Ir–O path. In contrast to the Ir foil, the WT contour plot of Ru_0.0738_-IrO_*x*_ is located at the maximum intensity, attributed to the Ir–Ir bond. However, no significant Ir–Ir paths are observed in Ru_0.0738_-IrO_*x*_, which is in agreement with the EXAFS fitting results. Furthermore, the XANES and extended EXAFS spectra of Ru_0.0738_-IrO_*x*_ and the reference samples were analyzed at the Ru K-edge. Figure 2g indicates that the oxidation state of Ru in Ru_0.0738_-IrO_*x*_ is marginally higher than 4+. This finding is consistent with the positive binding energy shifts observed in Ru XPS and supports the notion of Ru acting as an electron donor. Comparison of the FT-EXAFS spectra with those of RuO_2_ and Ru foil reveals that Ru preferentially forms Ru–O bonds with the coordinatively unsaturated oxygen sites on the amorphous IrO_*x*_ surface. The first-shell Ru–O coordination number (CN = 4.52) is significantly lower than the typical octahedral coordination number of Ir in IrO_*x*_ (≈6), indicating a distinct deviation of Ru’s coordination environment from the intrinsic lattice Ir coordination. Further analysis confirms the absence of discernible Ir–Ru scattering signals in the second shell, directly precluding the possibility of Ru substituting for Ir lattice sites. Concomitantly, the Ru–Ru coordination number approximates 1, demonstrating that Ru species exist as highly dispersed single atoms without significant aggregation. Collectively, these EXAFS results explicitly indicate that Ru does not incorporate into the IrO_*x*_ lattice to replace Ir atoms; instead, it anchors as single atoms on the IrO_*x*_ substrate surface, forming a distorted tetrahedral coordination environment with a coordination number of approximately 4 (Figures 2h and S8, Table S3).^46^ The lack of significant Ru–Ru scattering paths in the WT-EXAFS analysis (Figures 2g and S9) further implies that the Ru dopant is homogeneously dispersed at an atomic or near-atomic level within the IrO_*x*_ host structure without forming significant clusters. Based on the results of XPS and XAS, Ru doping reduces the valence and covalency of IrO_*x*_, which can promote the oxidation of water and accelerate the oxidation of the OER process.

### Electrocatalytic Performance of OER under 0.5 M H_2_SO_4_

To evaluate the OER electrocatalytic performance of Ru_0.0738_-IrO_*x*_, a three-electrode system was employed for electrochemical testing. The electrolyte was selected as 0.5 M H_2_SO_4_, the Hg/Hg_2_SO_4_ electrode was selected as a reference electrode, the Pt electrode (1 cm × 1 cm) was selected as a counter electrode, and the catalyst-coated carbon paper with a surface area of 1 cm^2^ was utilized as the working electrode. The catalyst loading on carbon paper was 0.285 mg cm^–2^. The LSV curves of Ru_*y*_-IrO_*x*_ samples with varying molar concentrations were compared with those of undoped IrO_*x*_, commercial IrO_2_, and commercial RuO_2_ samples (Figures 3a, S10a,b). The performance of the Ru_*y*_-IrO_*x*_ samples was found to be superior to that of the IrO_*x*_ sample, with the best performance observed in the Ru_0.0738_-IrO_*x*_ sample. Specifically, Ru_0.0738_-IrO_*x*_ exhibits the lowest peak-starting potential, with an overpotential of 225 mV at a current density of 10 mA cm^–2^, which was significantly lower than that of IrO_*x*_ (296 mV), C-IrO_2_ (348 mV), and C-RuO_2_ (359 mV). This suggests that Ru_0.0738_-IrO_*x*_ displays the most optimal OER catalytic activity. To assess intrinsic activity, mass activity and specific activity were calculated by normalizing the current to the catalyst loading, surface area, and electrochemically active surface area (ECSA), respectively, at an applied potential of 1.5 V (vs RHE) (Figures 3b and S11, Table S4).^17^ Ru_0.0738_-IrO_*x*_ demonstrated a mass activity of 86.07 mA mg_oxide_^–1^ and a specific activity of 0.0417 mA cm_ECSA_^–2^. These values substantially surpassed those of IrO_*x*_ (21.1 mA mg_oxide_^–1^ and 0.01671 mA cm_ECSA_^–2^), commercial IrO_2_ (6.78 mA mg_oxide_^–1^ and 0.01078 mA cm_ECSA_^–2^), and commercial RuO_2_ (39.36842 mA mg_oxide_^–1^ and 0.01817 mA cm_ECSA_^–2^). Notably, the turnover frequency (TOF value) of Ru_0.0738_-IrO_*x*_ at an overpotential of 300 mV reaches 0.67 s^–1^, which is 3.19, 16.75, and 22.3 times that of IrO*_x_* (0.21 s^–1^), commercial IrO_2_ (0.04 s^–1^), and commercial RuO_2_ (0.03 s^–1^), respectively (Table S5). The results indicated that Ru_0.0738_-IrO_*x*_ demonstrated the most optimal intrinsic OER activity, and doping of Ru significantly enhanced the catalytic activity of the IrO_*x*_ matrix. The microkinetics of the OER were investigated using Tafel analysis (Figures 3c and S10c).^14^ The Tafel slope for Ru_0.0738_-IrO_*x*_ was determined to be 98.1 mV dec^–1^, slightly lower than that of other Ru_*y*_-IrO_*x*_ samples but significantly lower than those of pure IrO_*x*_ (150.1 mV dec^–1^), commercial IrO_2_ (164.9 mV dec^–1^), and commercial RuO_2_ (131 mV dec^–1^). This lower Tafel slope suggests that Ru doping favorably alters the OER reaction mechanism, potentially modifying the rate-determining step (RDS) and enabling faster kinetics at lower overpotentials. Consequently, Ru_0.0738_-IrO_*x*_ exhibits the lowest overpotential when the catalyst is required to achieve the same current density. To gain insight into the origin of the OER activity, we performed a comparative analysis of the roughness factor (*R*_f_) and the ECSA by calculating the double electrical layer capacitance (*C*_dl_) (Figures 3d, S10d, and S12, Table S4). As shown in Figure 3d, the double electrical layer capacitance (*C*_dl_) of Ru_0.0738_-IrO_*x*_ (35.3 mF cm^–2^) was significantly higher than those of IrO_*x*_ (21.6 mF cm^–2^), C-IrO_2_ (4.76 mF cm^–2^), and C-RuO_2_ (5.2 mF cm^–2^). In general, a high *R*_f_ value is indicative of a catalyst with a large ECSA.^10^ According to the formula *R*_f_ = *C*_dl_/*C*_s_ (where *C*_s_ is the specific capacitance, which is usually equal to 0.06 mF cm^–2^),^28^ compared to IrO_*x*_ (*R*_f_ = 360), C-IrO_2_ (*R*_f_ = 79.3), and C-RuO_2_ (*R*_f_ = 86.7), Ru_0.0738_-IrO_*x*_ has an *R*_f_ value of 588.3. The ECSA is calculated by multiplying the *R*_f_ value by the geometric area of the electrode (1 cm^2^). So, the ECSA values for these catalysts are the same as *R*_f_. The larger ECSA indicates that the Ru_0.0738_-IrO_*x*_ electrocatalyst has more OER catalytic active sites. The surface area of Ru_0.0738_-IrO_*x*_ catalyst was 206.4 m^2^ g^–1^, which was larger than that of IrO_*x*_ (126.3 m^2^ g^–1^), C-IrO_2_ (27.8 m^2^ g^–1^), and C-RuO_2_ (30.4 m^2^ g^–1^). This finding aligns with its high BET surface area (206.4 m^2^ g^–1^, Figure S4), characteristic of its 2D nanosheet morphology.^8^ The impedances of Ru_*y*_-IrO_*x*_, IrO_*x*_, commercial IrO_2_, and commercial RuO_2_ were then evaluated by electrochemical impedance spectroscopy (EIS). The Nyquist plots for each catalyst (Figures 3e and S10e) were evaluated at 1.506 V (vs RHE), where the diameter of the semicircle correlates with the magnitude of the charge transfer resistance (*R*_ct_).^33^ The solution resistance (*R*_sol_) of all electrodes was about 2.21 Ω. The Ru_0.0738_-IrO_*x*_ electrode had the smallest semicircle diameter, resulting in the lowest charge transfer resistance (*R*_ct_) of about 7.5 Ω. This value is lower than the remaining molar ratios of Ru_*y*_-IrO_*x*_, IrO_*x*_ (11.79 Ω), C-IrO_2_ (15.59 Ω), and C-RuO_2_ (16.7 Ω). The results demonstrate that Ru_0.0738_-IrO_*x*_ exhibits the lowest interfacial resistance and the most rapid charge transfer rate, thereby enabling the most expedient reaction kinetics and the highest intrinsic activity in the OER process.

**Figure 3:**
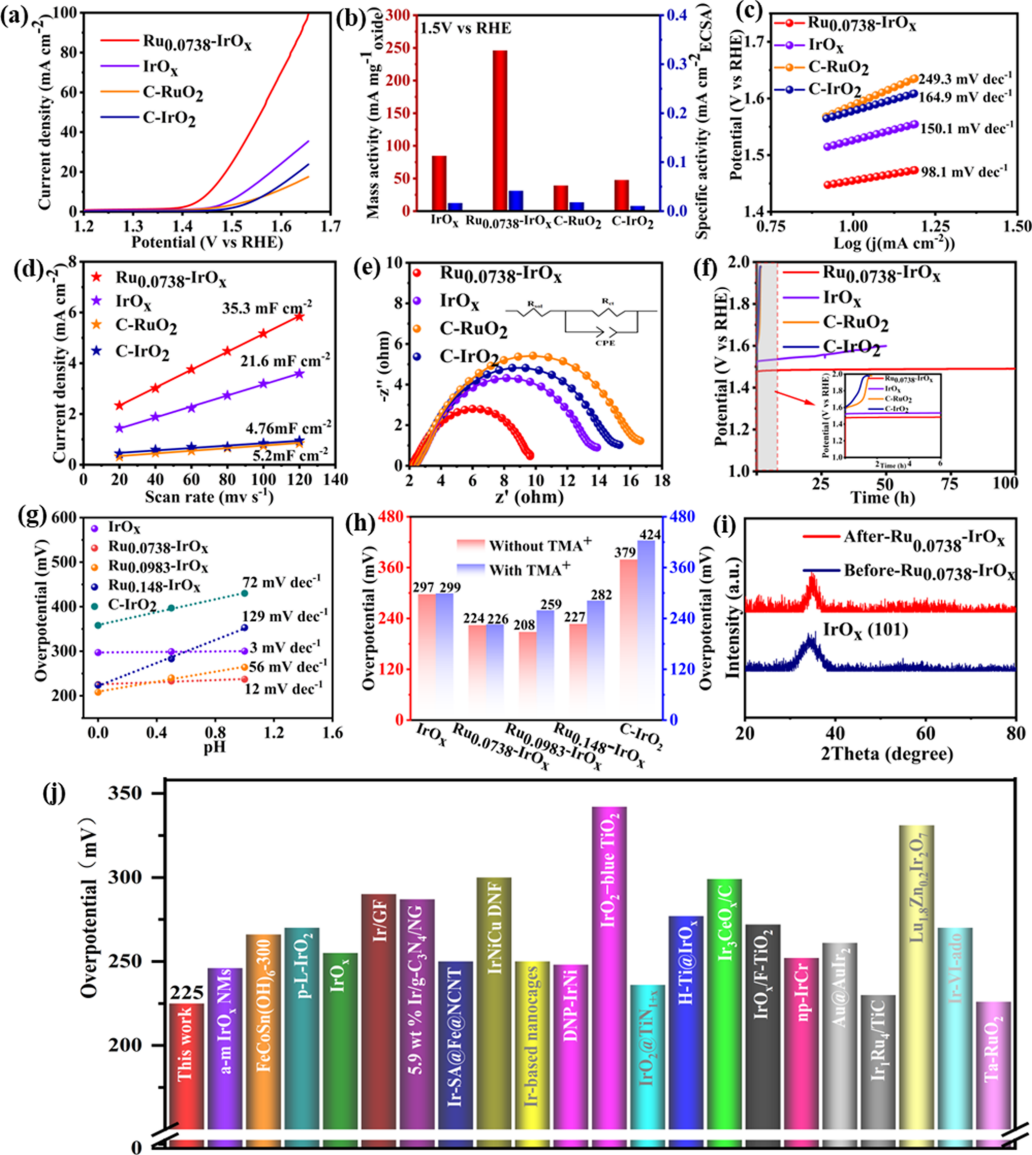
Electrochemical performance of Ru_0.0738_-IrO_*x*_, IrO_*x*_, C-IrO_2_, and C-RuO_2_ in an acidic medium (0.5 M H_2_SO_4_). (a) OER polarization curves. (b) Histogram comparing mass activity (normalized by catalyst loading) and intrinsic activity (normalized by ECSA) at 1.5 V vs RHE. (c) Tafel plots. (d) *C*_dl_ plots. (e) EIS plots. (f) Chronopotential test plots at 10 mA cm^–2^. (g) pH dependence on the overpotential at 10 mA cm^–2^ for IrO_*x*_, Ru_0.0738_-IrO_*x*_, Ru_0.0983_-IrO_*x*_, Ru_0.148_-IrO_*x*_, and C-IrO_2_ in H_2_SO_4_ with different pH values (0, 0.5, and 1). (h) Histograms of the overpotentials of IrO_*x*_, Ru_0.0738_-IrO_*x*_, Ru_0.0983_-IrO_*x*_, Ru_0.148_-IrO_*x*_, and C-IrO_2_ with and without the addition of TMAOH. (i) High-resolution XRD patterns of Ru_0.0738_-IrO_*x*_ both before and after the stability test. (j) Comparison of the overpotential of Ru_0.0738_-IrO_*x*_ with that of a recently reported Ir/Ru-based electrocatalyst at a current density of 10 mA cm^–2^ in an acidic medium.

Long-term stability is paramount for practical OER applications. Chronopotentiometric (V-t) tests showed that Ru_0.0738_-IrO_*x*_ maintained stable operation for 100 h at a current density of 10 mA cm^–2^ (Figure 3f). In contrast, both commercial IrO_2_ and commercial RuO_2_ exhibited a notable increase in overpotential and eventual deactivation after only 2 h stability testing. To study the effect of the concentration of Ru on the stability of IrO_*x*_, we carried out stability tests with different molar ratios of Ru_*y*_-IrO_*x*_ (Figure S10f). We found that Ru molar ratios exceeding 9.83% compromised the balance between activity and stability. This observation, coupled with XRD results showing RuO_2_ phase segregation at these higher Ru concentrations, suggests that the separated RuO_2_ phase may negatively impact the long-term stability. This could be attributed to the lattice oxygen mechanism (LOM) reported for RuO_2_ in acidic media, which can lead to structural degradation.^42^ Due to the poor stability of Ru_0.0983_-IrO_*x*_ at high Ru doping levels, Ru_0.0738_-IrO_*x*_ offers the optimal combination of high activity and robust stability. To determine the oxygen evolution efficiency of the OER process, the Faraday efficiency (FE) of the catalysts was evaluated. The FE of IrO_*x*_ and Ru_0.0738_-IrO_*x*_ were comparatively analyzed by assembling the device as shown in Figure S13a. As shown in Figure S13b,c and Table S6, the results show that the FE of Ru_0.0738_-IrO_*x*_ is very close to 100%. This indicates that the side reactions have a negligible effect on the results. Furthermore, Ru_0.0738_-IrO_*x*_ showed the lowest overpotential at a current density of 10 mA cm^–2^ (Figure 3j and Table S7), which surpassed the performance of previously reported Ir-based electrocatalysts under acidic conditions.

### Exploration of High-Performance Sources of Ru_0.0738_-IrO_*x*_

The operational stability of the OER catalysts is intrinsically linked to their reaction mechanism. Previous studies indicate that while the LOM can offer high initial activity, it often involves oxidation of lattice oxygen and subsequent structural degradation, leading to metal dissolution and accelerated catalyst deactivation.^6^ In contrast, the AEM, which proceeds via surface-adsorbed intermediates, is typically observed to exhibit superior catalytic stability.^47^ To ascertain the source of the high activity and excellent stability of Ru_0.0738_-IrO_*x*_, an investigation was conducted into the mechanism of the OER through the following approach. Typically, the oxygen evolution reaction can proceed via two primary pathways: the AEM involving a concerted proton–electron transfer step, and the LOM characterized by nonconcerted proton–electron transfer and pH-dependent catalytic activity.^48^ As illustrated in Figures 3g and S14, Ru_0.0983_-IrO_*x*_, Ru_0.148_-IrO_*x*_, and commercial IrO_2_ displayed pronounced pH-dependent behaviors, with Tafel slopes of 56, 129, and 72 mV dec^–1^, respectively. In stark contrast, Ru_0.0738_-IrO_*x*_ demonstrated negligible pH dependency, evidenced by a Tafel slope of only 12 mV dec^–1^. This discrepancy indicates that when the Ru doping molar ratios exceed 0.0983, a noncooperative proton–electron transfer step is observed in Ru_*y*_-IrO_*x*_, resulting in a transition from the AEM to the LOM pathway in the OER mechanism. Therefore, the OER processes of Ru_0.0983_-IrO_*x*_ and Ru_0.148_-IrO_*x*_ proceeded primarily through the LOM pathway, resulting in a rapid degradation rate at a current density of 10 mA cm^–2^ in an acidic medium (Figure S10f). In contrast, the exceptional stability of Ru_0.0738_-IrO_*x*_ was attributed to the AEM pathway. Tetramethylammonium hydroxide (TMAOH), a typical detector, contains TMA^+^ ions that are capable of forming strong bonds with oxygenated species, thereby competing with the LOM mechanism in the OER process and impeding the OER kinetics.^49^ The OER reaction mechanism was validated through a comparative analysis of the electrolyte with and without the addition of TMA^+^ ions. Figure 3h and Figure S15 illustrate that the catalytic activities of Ru_0.0983_-IrO_*x*_ and Ru_0.148_-IrO_*x*_ with added TMA^+^ ions were markedly diminished, indicating that the OER process was predominantly LOM-driven. Conversely, the catalytic activities of Ru_0.0738_-IrO_*x*_ and IrO_*x*_ exhibited no discernible decline before and after the introduction of TMA^+^ ions, indicating that the OER process was AEM-dominated. The aforementioned results indicate that the mechanism of the OER of Ru_0.0738_-IrO_*x*_ is predominantly AEM-driven. Subsequently, the stability of Ru_0.0738_-IrO_*x*_ was assessed after a 100 h chronopotentiometry test. To verify the structural stability of the Ru_0.0738_-IrO_*x*_ catalyst, XRD and STEM characterizations were performed subsequent to the stability test. The characterization (Figures 3i and S16) results indicated that the catalyst maintained its intact structure after testing, thereby confirming its excellent structural stability.

To investigate the origin of the excellent OER performance of the prepared catalysts, a series of DFT calculations and Ab Initio Molecular Dynamics simulations were performed based on the experimental observations. First, according to the characterization results, at low Ru doping concentration, Ru is most likely to be bound to the IrO_*x*_ surface as a single atom. Accordingly, DFT models for IrO_*x*_ as well as three distinct Ru-doped IrO_*x*_: Ru@IrO_*x*_-I, Ru@IrO_*x*_-II, and Ru@IrO_*x*_-III, were constructed based on plausible surface doping sites (Figure 4a). In Ru@IrO_*x*_-I, the Ru atoms are located at the centers of two Ir atoms, forming an Ir–Ru bond; in Ru@IrO_*x*_-II, the Ru atom was trapped by the exposed O atoms on the IrO_*x*_ surface, forming three Ru–O bonds; Finally, in Ru@IrO_*x*_-III, the Ru atom directly replaces an Ir atom on the surface of IrO_*x*_ and bonds with four surrounding O atoms. Calculation of Ru binding energies revealed that configurations Ru@IrO_*x*_-II and Ru@IrO_*x*_-III both exhibit negative binding energies, indicating thermodynamically favorable Ru incorporation via Ru–O bond formation on the IrO_*x*_ surface. Conversely, the binding energy of Ru@IrO_*x*_-I is positive, indicating that Ru–Ir bonds are challenging to immobilize on the IrO_*x*_ surface. This finding is consistent with our characterization results, which demonstrate the observation of Ru–O bonds by synchrotron radiation but not of Ru–Ir bonds.

**Figure 4:**
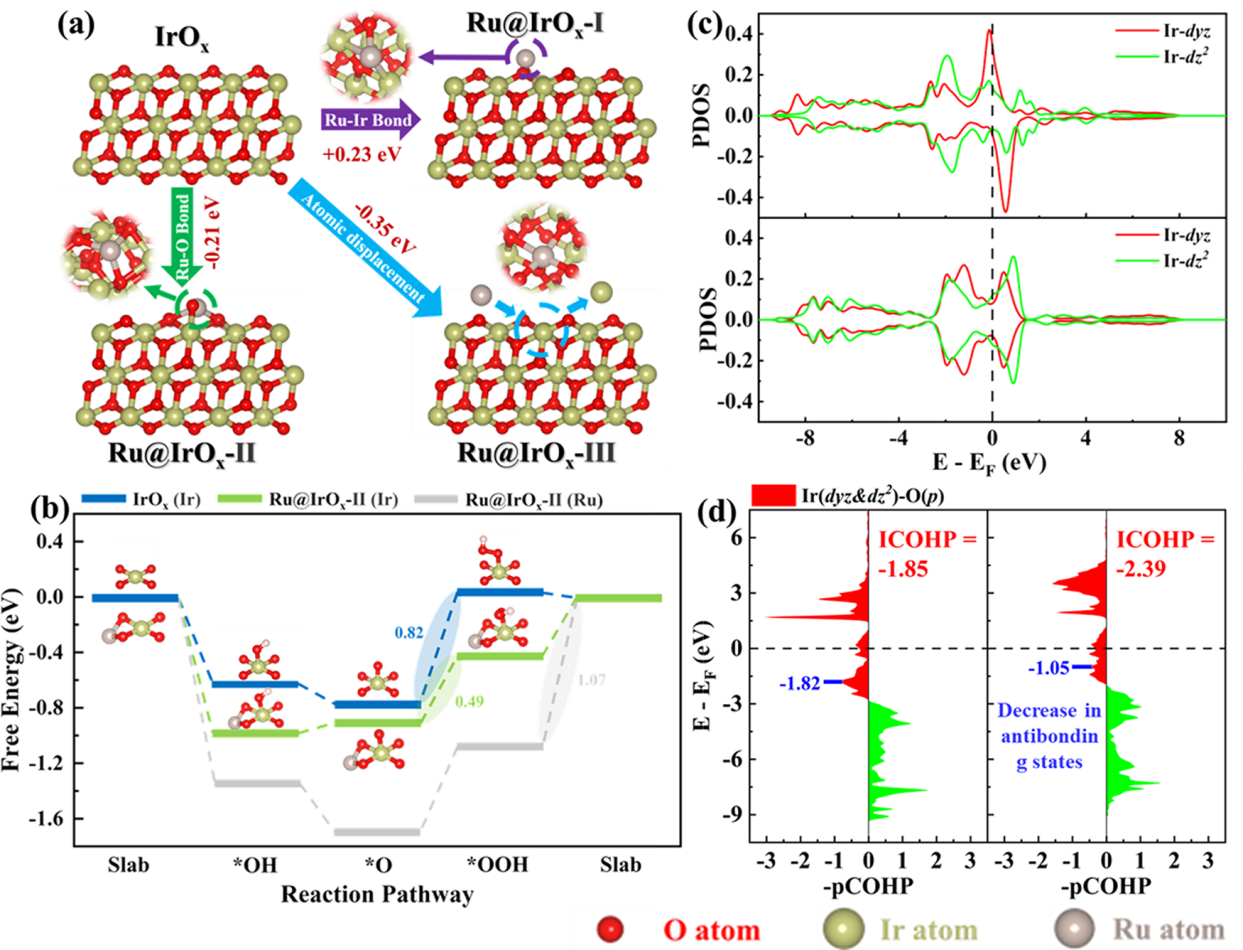
(a) IrO_*x*_ and the DFT model of IrO_*x*_ doped with Ru. (b) OER free energy diagram of Ir, Ru sites on IrO_2_, and Ru@IrO_*x*_-II (active sites in brackets, shaded areas indicate RDS). (c) PDOS of the Ir site on IrO_*x*_ (top panel) vs Ru@IrO_*x*_-II (bottom panel). (d) COHP analysis of Ir (*dyz* and *dz* ^2^)-O­(p) bonds in IrO_*x*_ (left panel) and Ru@IrO_*x*_-II (right panel) in the *OH intermediate state.

In this regard, subsequent studies of the OER process will be conducted at the Ir and Ru sites on IrO_*x*_, Ru@IrO_*x*_-II, and Ru@IrO_*x*_-III (Figure S17), based on the results of the stability discussion. For undoped IrO_*x*_, the entire OER will be carried out at its surface-exposed Ir site, where the rate-determining step (RDS) occurs at the *O to *OOH transition with a high transition energy barrier (Figures 4b and S18a). The catalytic efficiency of the OER was also not improved at the Ir and Ru sites exposed on the Ru@IrO_*x*_-III surface (Figure S19). In contrast, in Ru@IrO_*x*_-II, the energy barrier for the conversion of *O to *OOH on the Ir site was significantly reduced relative to the other systems with the site, although the reaction rate carried out at the Ru site in Ru@IrO_*x*_-II was still limited (Figures 4b and S18b).

Considering that the activity difference of IrO_*x*_ and Ru@IrO_*x*_-II may be due to the change in the electronic structure of the Ir site caused by the Ru doping, we further compared the projected density of states (PDOS), based on the adsorption position of the intermediate species in the Ir site, it is the *dyz* versus the *dz* ^2^ orbitals that are most likely to bind to the p orbitals of the O atom of their Ir sites. The results indicate that compared to IrO_2_, the *dyz* and *dz* ^2^ orbitals in Ru@IrO_*x*_-II shift upward toward the Fermi level (Figure 4c, blue shaded area). This shift causes the *dyz* and *dz* ^2^ orbitals at the Ir sites to lose electrons when forming bonds with the p orbitals of O atoms (denoted Ir­(*dyz* and *dyz* ^2^)–O­(p) bond in the case of *OH) in the reaction intermediates, as the antibonding orbitals become occupied, and the antibonding states of the Ir­(*dyz* and *dyz* ^2^)–O­(p) bonds below the Fermi level are also reduced (Figure 4d). This shift leads to the loss of electrons from the antibonding orbitals when the *dyz* and *dz* ^2^ orbitals at the Ir sites couple with the p orbitals of O atoms (denoted Ir­(*dyz* and *dyz* ^2^)–O­(p) bond in the case of *OH) in the reaction intermediates. As a result, the antibonding state of the Ir­(*dyz* and *dyz* ^2^)–O­(p) bond is correspondingly reduced below the Fermi level (Figure 4d). Based on the above discussion, the synergistic effect of a small amount of Ru doping on the modification of the electronic structure of IrO_*x*_ moderately enhances the adsorption of intermediate species at the Ir site, which in turn drastically reduces the energy barriers required for the occurrence of the OER.

Finally, at high Ru doping concentrations, Ru monatomic will agglomerate above IrO_*x*_ and be oxidized to form RuO_2_. In this context, we used AIMD simulations to further observe the thermal stability of this system (RuO_2_@IrO_*x*_) (Figure S20). From the structural changes and energies during the 10 ps simulation, although the IrO_*x*_ structure remains intact at 5000 and 10 000 fs compared to that at 0 fs, the Ru–O bond in the upper RuO_2_ is broken, leading to a severe distortion of the geometry, which explains why the energy oscillation of the RuO_2_@IrO_*x*_ system is larger in amplitude during the 10 ps simulation. Therefore, RuO_2_@IrO_*x*_ formed at high Ru doping concentration is prone to reconfiguration during the reaction process, making it difficult to maintain the thermal stability of its system.

## Conclusions

In conclusion, we have established a successful design strategy for a high-performance acidic OER catalyst by incorporating Ru dopants into an ultrathin, amorphous IrO_*x*_ nanosheet matrix. This approach effectively overcomes the persistent activity-stability trade-off inherent to iridium-based catalysts. A comprehensive investigation combining XPS and XAS spectroscopy and DFT provided a deep understanding of the synergistic effects at play. We determined that atomically dispersed Ru dopants induce charge transfer to adjacent Ir sites, which precisely modulates the electronic structure of the Ir active centers. This electronic engineering accomplishes two critical tasks simultaneously: it significantly lowers the activation energy barrier for the rate-limiting *O-to-*OOH transformation, and it steers the reaction pathway toward the stable AEM, thereby suppressing the degradative LOM. The direct consequence of this optimized structure and mechanism is a catalyst with superior performance. The engineered Ru-doped amorphous IrO_*x*_ nanosheets exhibit remarkable OER activity and exceptional durability in harsh acidic conditions, significantly outperforming conventional benchmarks. This work not only presents a highly effective catalyst but also establishes a clear design principle: leveraging a chemically stable amorphous host while using atomic-level dopants to tune the electronic structure and reaction pathway is a powerful strategy for developing next-generation catalysts for challenging electrochemical processes.

## Methods

### Synthesis of IrO_*x*_ NSs

IrCl_3_ solution was prepared in a specific ratio (Ir content: 35 mg/mL). 500 μL of the IrCl_3_ solution was transferred to an 8 mL glass vial and sonicated. Then, 5 g of sodium nitrate (NaNO_3_) was weighed into a 25 mL beaker. The IrCl_3_ solution in the glass vial was transferred to the surface of NaNO_3_ and dried in a drying oven at 60 °C for 30 min. The dried reagent mixture was transferred to a muffle furnace at 350 °C for 35 min, removed, and cooled to room temperature. The products were collected in 5 mL centrifuge tubes by sonication and centrifugation. Finally, the centrifuge tube was dried under vacuum at 60 °C for 12 h.

### Synthesis of Ru_*y*_-IrO_*x*_ NSs

IrCl_3_ and RuCl_3_ solutions were prepared in a specific ratio (Ir content: 35 mg/mL; Ru content: 92.5 mg/mL). Then, a certain amount of IrCl_3_ solution (570 μL) and RuCl_3_ were mixed in specific proportions (the molar ratios of Ir/Ru are 1:0.0328, 1:0.0492, 1:0.0738, 1:0.0983, and 1:0.148, respectively) in an 8 mL glass vial and sonicated to achieve uniform mixing. 5 g of NaNO_3_ was weighed into a 25 mL beaker, and the mixed solution was added to the NaNO_3_ surface. The beaker was then dried in an oven at 60 °C for 30 min. After the dried reagent mixture was transferred to a muffle furnace at 350 °C for 35 min, the samples were removed and cooled to room temperature. The samples were collected in 5 mL centrifuge tubes by sonication and centrifugation. Finally, the samples were dried under a vacuum at 60 °C for 12 h.

### Structural Characterization

XRD patterns were obtained using a Panacor Empyren instrument with a Cu Kα radiation source and a scanning range of 20–80°. Inductively coupled plasma-mass spectrometry (ICP-MS) was tested using a U.S.-Aglient-5110 (OES) unit. To characterize the morphology and nanostructure of the samples, focused ion beam scanning electron microscopy (SEM, TESCAN-AMBER) and a JEOL ARM 300 double Cs-corrected TEM operated at 300 kV were employed. The specific surface area and pore size of the samples were analyzed using a BET-USA Micromeritics ASAP 2460. To determine the surface compositions and valence states of the materials, XPS (Shimadzu AXIS SUPRA+) was utilized. The characterization of oxygen vacancy defects was conducted through paramagnetic resonance spectroscopy (EPR) (BRUKER EMXPLUS). The morphology of the sample (including the distribution of the thickness) was characterized by atomic force microscopy (AFM) (BRUKER EDGE AFM).

### Electrochemical Characterization

Electrochemical performance testing: A three-electrode system (electrochemical workstation, CHI 760E, Shanghai, Chenhua, China) was employed for electrochemical performance testing. The platinum electrode (1 cm × 1 cm) was selected as the counter electrode, the carbon paper loaded with the catalyst was selected as the working electrode, the Hg/Hg_2_SO_4_ electrode was selected as the reference electrode, and the electrolyte was selected as a 0.5 M H_2_SO_4_ aqueous solution. The catalyst activation process was conducted via cyclic voltammetry (CV) at a scan rate of 100 mv s^–1^. The capacitance of the bilayer (*C*_dl_) was determined by measuring CV at incremental scan rates between 20 and 120 mV s^–1^ (in 20 mV s^–1^ sequential increments). A linear scanning voltammetry (LSV) measurement was conducted at a scan rate of 5 mV s^–1^ with 95% *iR* compensation applied. Electrochemical impedance spectroscopy (EIS) was conducted within a frequency range of 10 kHz–0.1 Hz. The chronopotentiometry (*V*–*t* curve) at a current density of 10 mA cm^–2^ was selected to evaluate the stability of the system.

### Theoretical Calculations

All first-principles calculations were implemented using VASP (Vienna Ab initio Simulation Package) version 6.3.0. The exchange-correlation generalization is the Perdew–Burke–Ernzerhof (PBE) generalization under the generalized gradient approximation (GGA), and the electron–ion interactions are described by the projective affine-added plane-wave (PAW) pseudopotential. The truncation energy of the plane-wave basis group was set to 520 eV, and a 2 × 2 × 1 *K*-point grid was used for the Brillouin zone integration. The convergence criteria for the energy and force are set to 1 × 10^–6^ and 0.01 eV/Å, respectively, during the structural optimization process. To accurately describe the van der Waals interactions, the DFT-D3 correction method is used in the calculations. In this case, the vacuum layer was set to 15 Å to avoid interactions between the periodic structures. The crystal orbital Hamiltonian placement (COHP) method was then used to analyze the bonding and antibonding properties of chemical bonds between atoms. The COHP analysis was implemented by LOBSTER software, and the chemical bond strength was measured by integrating the COHP curve (ICOHP). In addition, the thermal stability of the model was tested by ab initio molecular dynamics (AIMD) simulations. Specifically, the AIMD simulations were executed under the NVT synthesis paradigm, employing a Nosé-Hoover heat bath at a controlled temperature of 500 K. The time step was set to 1 fs, and the total simulation duration was 10 ps.

## Supplementary Material



## Notes

Methods; structural characterization; electrochemical characterization; theoretical calculations; Ru content of catalysts measured by ICP-AES/MS; XRD patterns of Ru*_y_*-IrO*_x_* (PDF)
