# Supplementary material for: Synergistic Ruthenium-Doped Amorphous IrO x Matrix for Robust Oxygen Evolution
Source: ACS Appl Mater Interfaces. 2025 Sep 19;17(39):54839–49. doi: 10.1021/acsami.5c13101 (PMC12755195; doi:10.1021/acsami.5c13101)
Supplement: Supplementary file 1 [file am5c13101_si_001.pdf]

Supporting Information

Synergistic Ruthenium Doped Amorphous IrO_x Matrix for Robust Oxygen Evolution

Jiandong Hu,[1 ‡] Yangfan Liu,[1 ‡] Yanlin Jia,[1] Ziye Li,[1] Haowei Yang,[1] Yang Wang,[1] Wenhui Luo,[1] Zhi Liang Zhao,[2] Yejun Li,[1] Yong Pang[1] and Qi Wang[3]**

1. School of Materials Science and Engineering, Central South University, Changsha, Hunan 410083, P.R. China,

   Email: thgink@126.com

2. Foshan Xianhu Laboratory, National energy key laboratory for new hydrogen-ammonia energy technologies, Foshan, Guangdong 528200, P.R. China

3. Department of Materials Science and Engineering, City University of Hong Kong, Hong Kong 999077, China

Email: qwang422@cityu.edu.hk

[‡]: These authors have contributed equally to this work

Methods

The Chemical Raw Materials

Iridium chloride ($IrCl_3$, 99%, Adamas), anhydrous ruthenium chloride ($RuCl_3$, 37%, Adamas), and Nafion (5 wt %) were procured from Shanghai Titan Technology. Sulfuric acid (H_2SO_4, 98%), anhydrous ethanol, and sodium nitrate (99.0%) were procured from Sinopharm Chemical Reagent. Carbon paper was supplied by Shanghai Huayu Instrumentation. All reagents were of analytical grade and did not require further purification.

Synthesis of IrO_x NSs

$IrCl_3$ solution was prepared in a specific ratio (Ir content: 35 mg/mL). 500 μL of the $IrCl_3$ solution was transferred to an 8 mL glass vial and sonicated. Then 5 g of sodium nitrate ($NaNO_3$) was weighed into a 25 mL beaker. The $IrCl_3$ solution in the glass vial was transferred to the surface of the $NaNO_3$ and dried in a drying oven at 60 °C for 30 min. The dried reagent mixture was transferred to a muffle furnace at 350 °C for 35 min, removed, and cooled to room temperature. The products were collected in 5 mL centrifuge tubes by sonication and centrifugation. Finally, the centrifuge tube was dried under vacuum at 60 °C for 12 h.

Synthesis of Ru_y-IrO_x NSs

$IrCl_3$ and $RuCl_3$ solutions were prepared in a specific ratio (Ir content: 35 mg/mL) (Ru content: 92.5 mg/mL). Then, a certain amount of $IrCl_3$ solution (570 μL) and $RuCl_3$ were mixed in specific proportions (The Ir/Ru molar ratios are set as 1:0.0328, 1:0.0492, 1:0.0738, 1:0.0983, and 1:0.148, respectively. This series of ratios is derived from a systematic gradient design: starting with an initial Ru/Ir molar ratio of 0.0328 for the first catalyst, each subsequent ratio is obtained by a 1.5-fold incremental increase

of the prior Ru content relative to Ir) in an 8 mL glass vial and sonicated to achieve uniform mixing. 5 g of $NaNO_3$ (The total mass of precursors was calculated using the maximum Ru/Ir molar ratio (0.148). Referring to the optimal $NaNO_3$-to-precursor ratio of approximately 13:1 for molten salt synthesis, the dosage of $NaNO_3$ was determined as 5 g. Consequently, a consistent amount of 5 g $NaNO_3$ was employed for the synthesis of all Ru-IrO_x samples) was weighed into a 25 mL beaker and the mixed solution was added to the $NaNO_3$ surface. The beaker was then dried in an oven at 60°C for 30 min. After transferring the dried reagent mixture to a muffle furnace at 350°C for 35 min, the samples were removed and cooled to room temperature. The samples were collected in 5 mL centrifuge tubes by sonication and centrifugation. Finally, the samples were dried under vacuum at 60°C for 12 h.

Structural Characterization

X-ray diffraction (XRD) patterns were obtained using a Panacor Empyren instrument with a Cu Kα radiation source and a scanning range of 20° to 80°. Inductively coupled plasma mass spectrometry (ICP-MS) was tested using a U.S.-Aglient-5110 (OES) unit. To characterize the morphology and nanostructure of the samples, focused ion beam scanning electron microscopy (SEM, TESCAN-AMBER) and JEOL ARM 300 double Cs-corrected TEM operated 300 kV were employed. The specific surface area and pore size of the samples were analyzed using a Brunauer-Emmett-Teller (BET)-USA Micromeritics ASAP 2460. To determine the surface compositions and valence states of the materials, X-ray photoelectron spectroscopy (XPS) (Shimadzu AXIS SUPRA+) was utilized. The characterization of oxygen vacancy defects was conducted through

paramagnetic resonance spectroscopy (EPR) (BRUKER EMXPLUS). The morphology of the sample (including the distribution of thickness) was characterized by atomic force microscopy (AFM) (BRUKER EDGE AFM).

Electrochemical Characterization

Preparation of working electrode: A solution comprising 2 mg of catalyst and 10 μL of Nafion (5 wt %) was prepared, along with a further 700 μL of ethanol, in a total volume of 710 μL. The samples were thoroughly mixed for 30 min using an ultrasonic machine. Subsequently, 100 μL of ink was transferred to a carbon paper surface with an area of 1 cm^2 (catalyst loading ~285 μg cm^{-2}), followed by drying with an infrared lamp for use.

Electrochemical performance testing: A three-electrode system (electrochemical workstation, CHI 760E, Shanghai Chenhua, China) was employed for the electrochemical performance testing. The platinum electrode (1 × 1 cm) was selected as the counter electrode, the carbon paper loaded with the catalyst was selected as the working electrode, the Hg/Hg_2SO_4 electrode was selected as the reference electrode, and the electrolyte was selected as a 0.5 M H_2SO_4 aqueous solution. The catalyst activation process was conducted via cyclic voltammetry (CV) at a scan rate of 100 mV s^{-1}. The capacitance of the bilayer (C_{dl}) was determined by measuring CV at incremental scan rates between 20 mV s^{-1} and 120 mV s^{-1} (in 20 mV s^{-1} sequential increments). A linear scanning voltammetry (LSV) measurement was conducted at a scan rate of 5 mV s^{-1} with 95% iR compensation applied. Electrochemical impedance spectroscopy (EIS) was conducted within a frequency range of 10 kHz to 0.1 Hz. The chronopotentiometry

(V-t curve) at a current density of 10 mA cm^{-2} was selected to evaluate the stability of the system.

The Faraday efficiency (FE) was measured using the drainage method as described in the experimental method below:

A stability test was carried out by applying a constant current of 10 mA to the H-type electrolysis cell. The test was started when the electrodes were stable. The test lasted for 1.5 h and the amount of oxygen collected was recorded every 18 min. The same procedure was repeated 5 times and averaged to calculate the final FE result.

The FE and theoretical oxygen production were determined using the following formula (S(1) and S(2)).

$$FE(O_2,\%) = \frac{V_{O_2} \times n \times F}{V_m \times i \times t} \times 100 \qquad S(1)$$

$$n(theoretical\ O_2, mol) = \frac{i \times t}{n \times F} \qquad S(2)$$

Where V_{O2} is the volume of actual oxygen production (L), n is the number of electrons transferred during oxygen precipitation (n=4), F is the Faraday's constant (F=96485 C·mol^{-1}), V_m is the molar volume of the gas at room temperature (V_m=22.4 L·mol^{-1}), i is the applied current (i=0.01 A), t is the time of the reaction (s).

The TOF was calculated based on the method reported in previous work[1]. This calculation assumes 100% Faradaic efficiency, the specific calculation formulas (S(3) and S(4)) are as follows:

$$TOF = \frac{N_{O_2}}{N_{metal}} \qquad S(3)$$

N_{O_2} is the number of O_2 turnovers, calculated using the following formula:

$$N_{O_2} = \frac{j\left(\frac{A}{cm^2}\right) \times Scm^2{}_{oxide} \times 1\frac{C}{s} \times 1mol\ e^-}{96{,}485C \times 4e^-} \times N_A$$

S(4)

A is the surface area of electrode, N_A is Avogadro constant (6.02×10^{23} mol^{-1}) and j is the measured current density. Besides, the number of metal sites (N_{metal}) only calculated the metal (Ir) number sites in tested catalyst.

Theoretical calculations

All first-principles calculations were implemented using VASP (Vienna Ab initio Simulation Package) version 6.3.0. The exchange-correlation generalization is the Perdew-Burke-Ernzerhof (PBE) generalization under the generalized gradient approximation (GGA), and the electron-ion interactions are described by the projective affine-added plane wave (PAW) pseudopotential. The truncation energy of the plane-wave basis group has been set to 520 eV, and a 2 × 2 × 1 K-point grid has been used for the Brillouin zone integration. The convergence criteria for the energy and force are set to 1×10^{-6} and 0.01 eV/Å, respectively, during the structural optimization process. To accurately describe the van der Waals interactions, the DFT-D3 correction method is used in the calculations. In this case, the vacuum layer was set to 15 Å to avoid interactions between periodic structures. The crystal orbital Hamiltonian placement (COHP) method was then used to analyze the bonding and antibonding properties of chemical bonds between atoms. The COHP analysis was implemented by LOBSTER software and the chemical bond strength was measured by integrating the COHP curve (ICOHP).In addition, the thermal stability of the model was tested by ab initio molecular dynamics (AIMD) simulations. Specifically, the AIMD simulations were executed under the NVT synthesis paradigm, employing a Nosé-Hoover heat bath with a controlled temperature of 500 Kelvin. The time step was set to 1 fs, and the total simulation duration was 10 ps.

Table S1. Ru content of catalysts measured by ICP-AES/MS.

Catalysts	Ru-element content (%)
IrO_x	0
$Ru_{0.0328}$-IrO_x	2.327%
$Ru_{0.0492}$-IrO_x	4.378%
$Ru_{0.0738}$-IrO_x	6.913%
$Ru_{0.0983}$-IrO_x	9.597%
$Ru_{0.148}$-IrO_x	14.545%

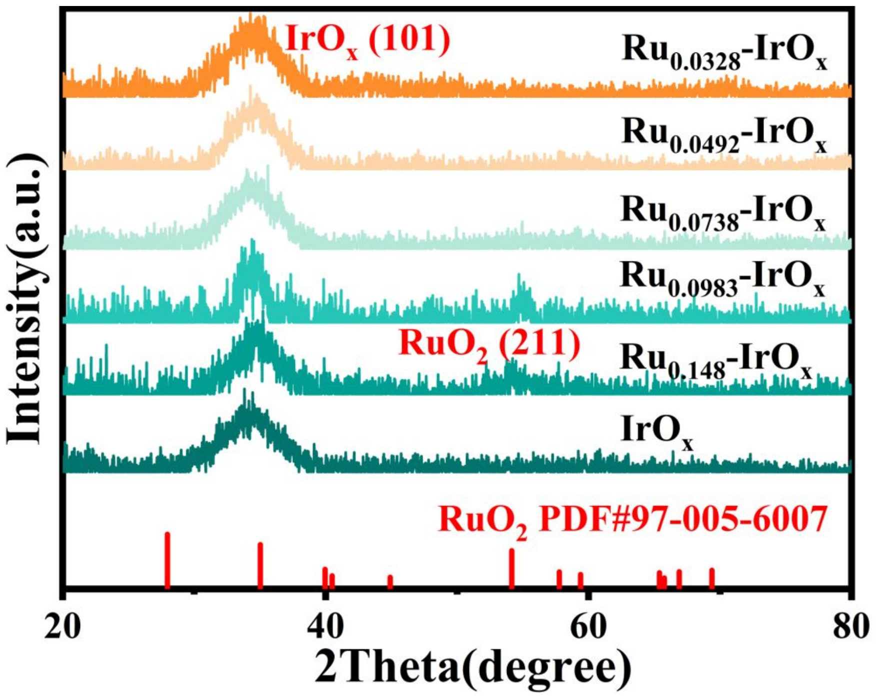

Figure S1. XRD patterns of Ru_y-IrO_x.

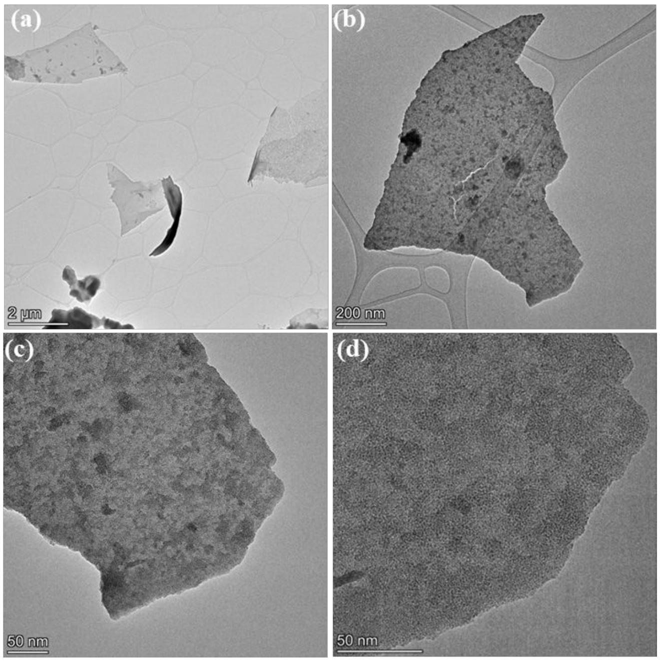

Figure S2. (a)-(d) Low magnification STEM images of $Ru_{0.0738}$-IrO_x (2μm, 200nm, 50nm, and 50nm).

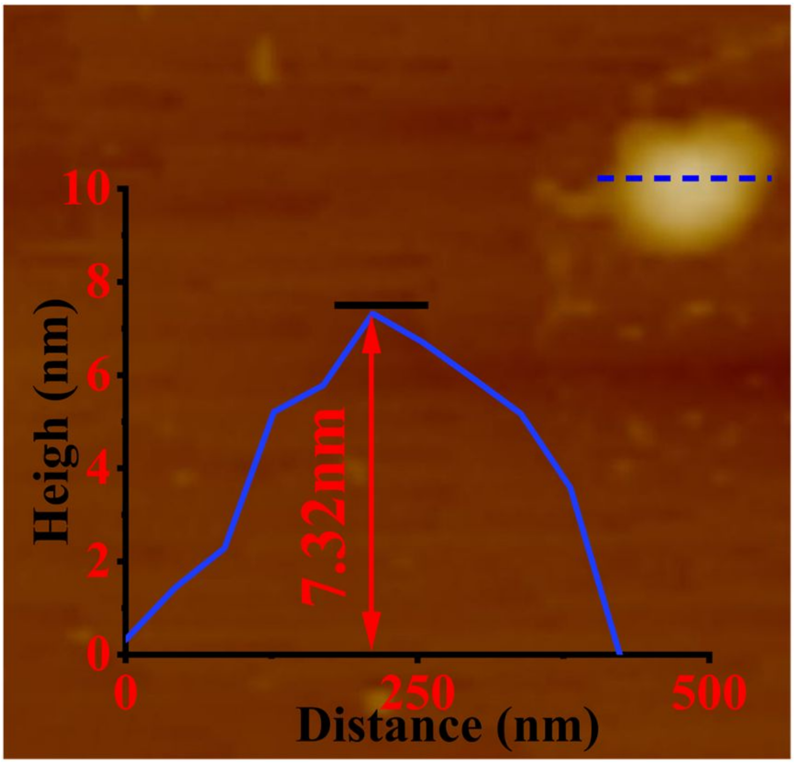

Figure S3.AFM image of IrO_x.

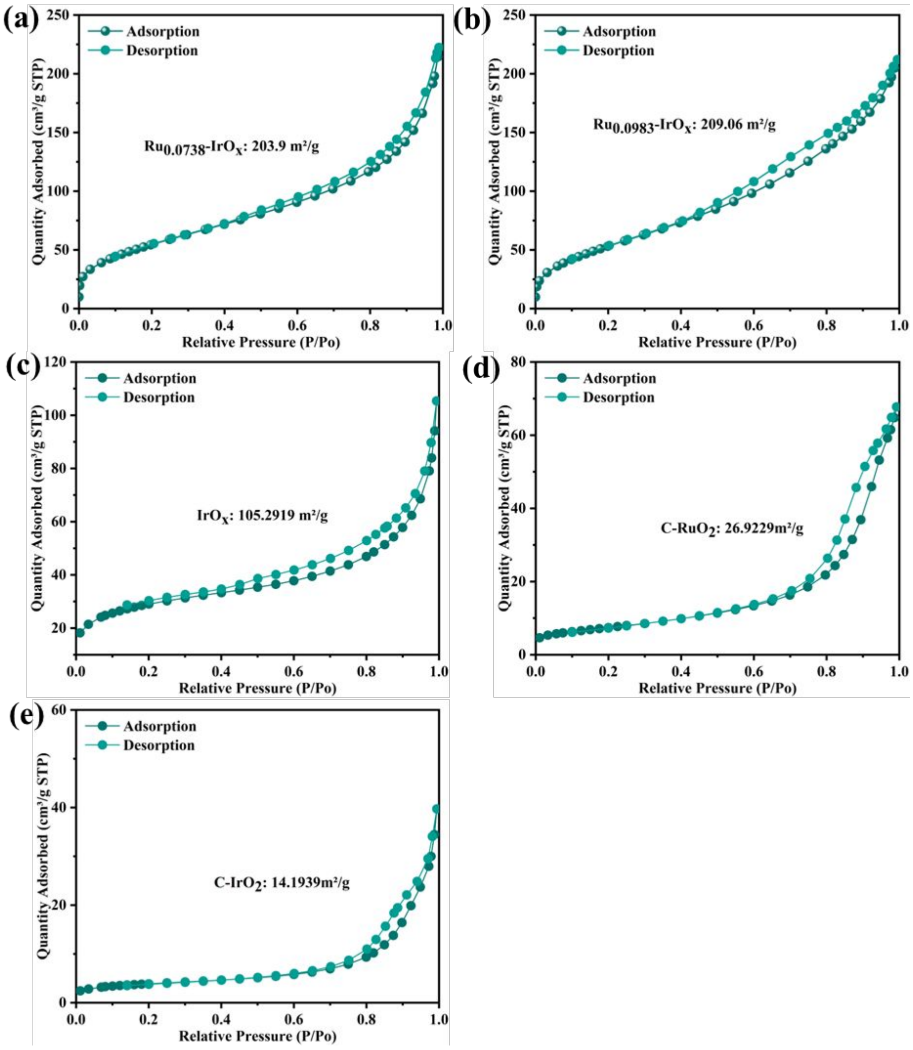

Figure S4. (a) The isotherm of N_2 adsorption and desorption on $Ru_{0.0738}$-IrO_x catalyst. (b) The isotherm of N_2 adsorption and desorption on $Ru_{0.0983}$-IrO_x catalyst. (c) The isotherm of N_2 adsorption and desorption on IrO_x catalyst. (d) The isotherm of N_2 adsorption and desorption on C-RuO_2 catalyst. (e) The isotherm of N_2 adsorption and desorption on C-IrO_2 catalyst.

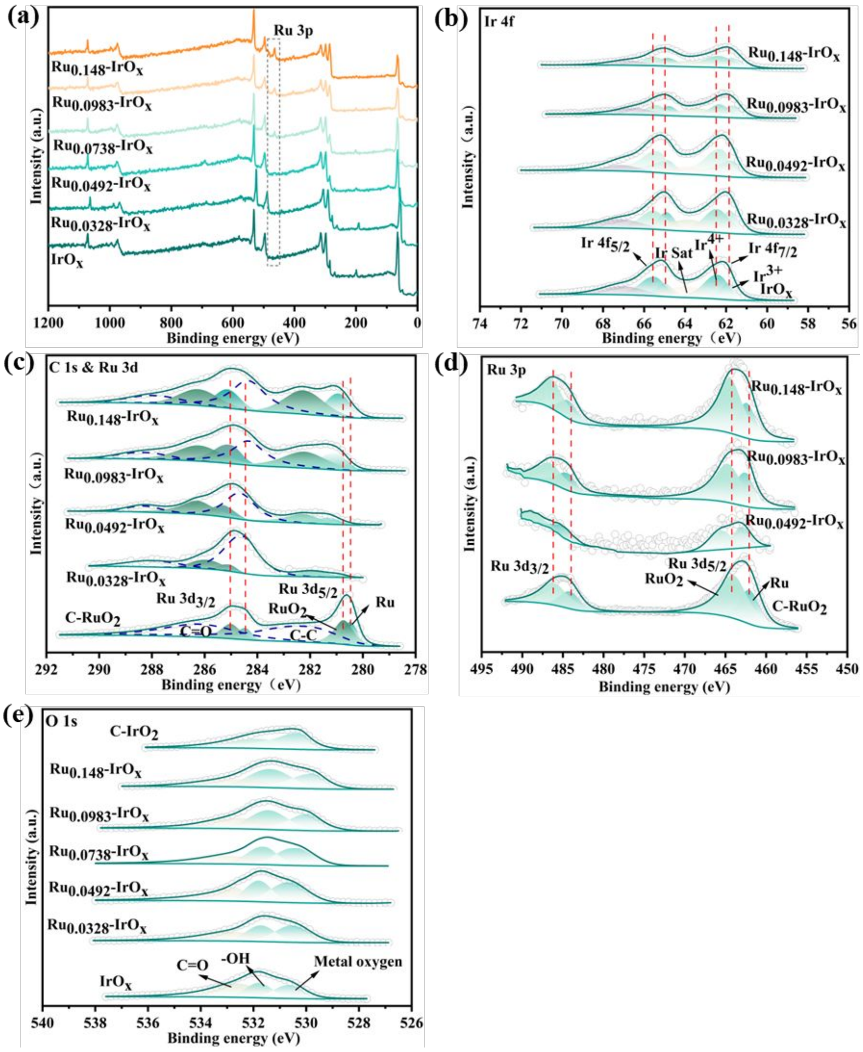

Figure S5. (a) The high-resolution XPS full-spectrum spectra of IrO_x, $Ru_{0.0328}$-IrO_x, $Ru_{0.0492}$-IrO_x, $Ru_{0.0738}$-IrO_x, $Ru_{0.0983}$-IrO_x, and $Ru_{0.148}$-IrO_x. (b) High-resolution X-ray photoelectron spectra of Ir 4f of IrO_x, $Ru_{0.0328}$-IrO_x, $Ru_{0.0492}$-IrO_x, $Ru_{0.0983}$-IrO_x, and $Ru_{0.148}$-IrO_x. (c) High-resolution X-ray photoelectron spectra of C 1s & Ru 3d of C-RuO_2, $Ru_{0.0328}$-IrO_x, $Ru_{0.0492}$-IrO_x, $Ru_{0.0983}$-IrO_x, and $Ru_{0.148}$-IrO_x. (d) High-resolution X-ray photoelectron spectra of Ru 3p of C-RuO_2, $Ru_{0.0492}$-IrO_x, $Ru_{0.0983}$-IrO_x, and $Ru_{0.148}$-IrO_x. (e) High-resolution X-ray photoelectron spectra of O 1s for IrO_x, $Ru_{0.0328}$-IrO_x, $Ru_{0.0492}$-IrO_x, $Ru_{0.0983}$-IrO_x, $Ru_{0.148}$-IrO_x, and C-IrO_2.

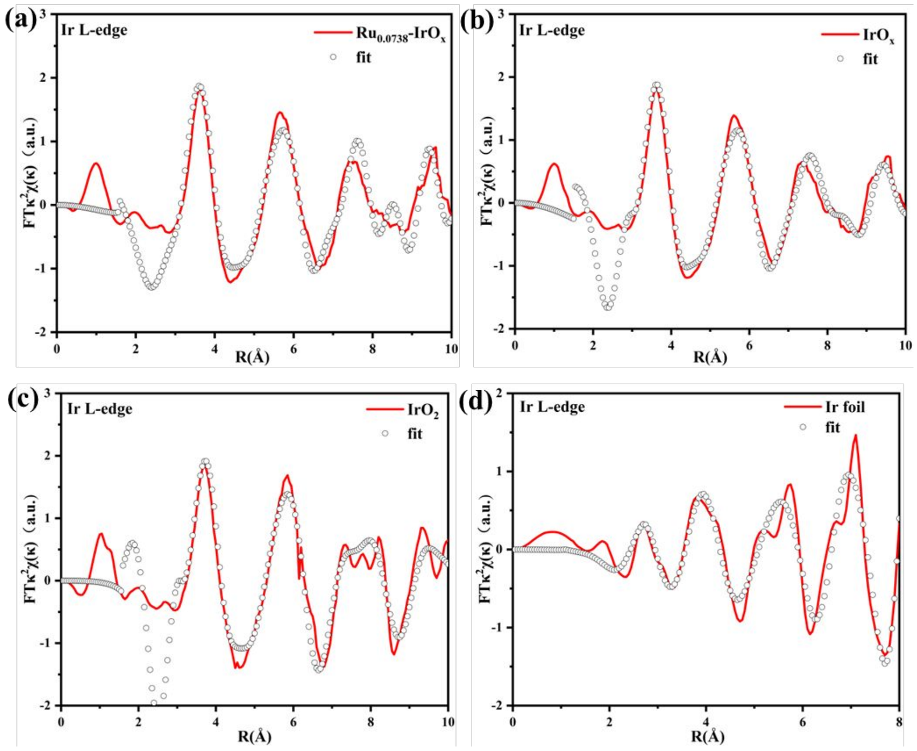

Figure S6. The EXAFS Ir L-space fitting results of (a) $Ru_{0.0738}$-IrO_x, (b) IrO_x, (c) IrO_2, and (d) Ir foil.

Table S2. EXAFS fitting parameters of Ir foil, IrO_2, IrO_x, and $Ru_{0.0738}$-IrO_x. (S_0^2 = 0.88).

Sample	Path	C.N.	R(Å)	σ^2(10^{-3}xÅ^2)	ΔE(eV)	R factor
**Ir foil**	Ir-Ir_1	10.6	2.743	4.42	4.48	0.0057
	Ir-Ir_2	0.096	3.849	3.48	4.48	
**IrO_2**	Ir-O	5.23	1.968	2.15	9.65	0.024
	Ir-Ir	10.03	3.139	12.55	9.65	
**IrO_x**	Ir-O	5.54	1.979	4.54	9.035	0.043
	Ir-Ir	9.13	3.092	6.49	9.035	
**$Ru_{0.0738}$-IrO_x**	Ir-O	5.52	1.995	4.3	9.291	0.039
	Ir-Ir	7.014	3.089	13.03	9.291	

Note:

R-factor is the sum of the squares of the differences between the data and the fit at each data point, divided by the sum of the squares of the data at each corresponding point.

N is the coordination number.

S_0^2 is the amplitude reduction factor.

σ^2 is the mean square relative displacement. It is a measurement of static and/or thermal disorder terms.

α is the change of the interatomic distance relative to the initial path length.

R is the path length.

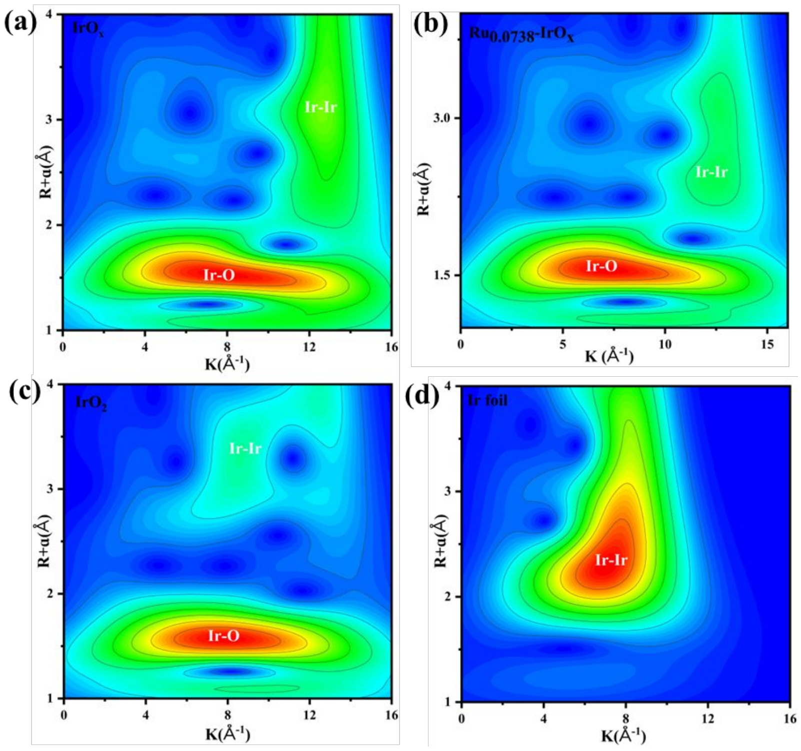

Figure S7. Wavelet transforms of k^2-weighted EXAFS spectra of the Ir K-edge for (a) IrO_x, (b) $Ru_{0.0738}$-IrO_x, (c) IrO_2, and (d) Ir foil.

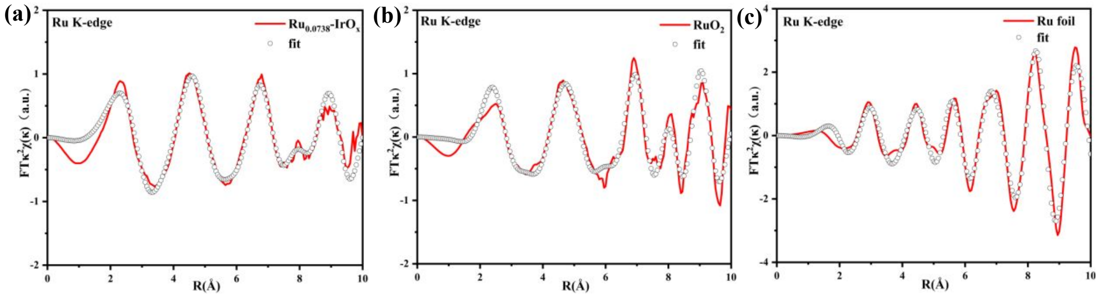

Figure S8. The EXAFS Ru k-space fitting results of (a) $Ru_{0.0738}$-IrO_x, (b) RuO_2, and (c) Ru foil.

Table S3. EXAFS fitting parameters of Ru foil, RuO_2, and $Ru_{0.0738}$-IrO_x. (S_0^2 = 0.88)

Sample	Path	C.N.	R(Å)	$\sigma^2(10^{-3}$xÅ$^2)$	ΔE(eV)	R factor
**Ru foil**	Ru-Ru_1	9.02	2.672	3.13	5.25	0.0147
	Ru-Ru_2	8.31	3.766	7.93	5.25	
**RuO_2**	Ru-O	5.64	1.955	0.12	0.047	0.0215
	Ru-Ru	5.28	3.179	14.45	0.047	
**$Ru_{0.0738}$-IrO_x**	Ru-O	4.52	1.989	1.78	1.108	0.0143
	Ru-Ru	1.76	3.177	2.61	1.108	

Note:

R-factor is the sum of the squares of the differences between the data and the fit at each data point, divided by the sum of the squares of the data at each corresponding point.

N is the coordination number.

S_0^2 is the amplitude reduction factor.

σ^2 is the mean square relative displacement. It is a measurement of static and/or thermal disorder terms.

α is the change of the interatomic distance relative to the initial path length.

R is the path length.

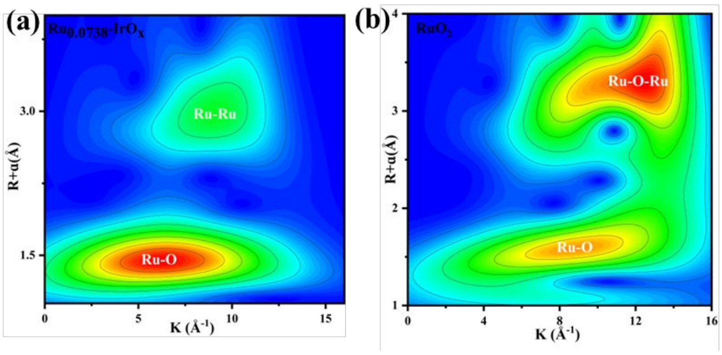

Figure S9. Wavelet transforms of k^2-weighted EXAFS spectra of the Ru K-edge for (a) $Ru_{0.0738}$-IrO_x, (b) RuO_2, and (c) Ru foil.

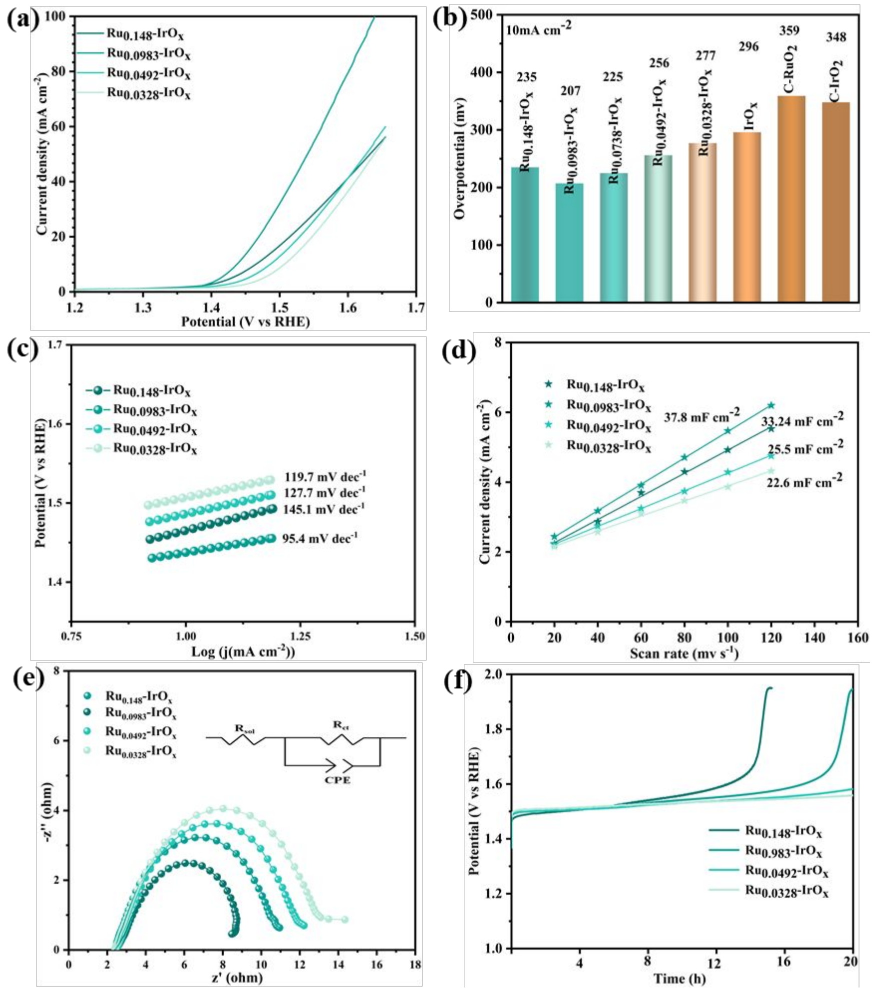

Figure S10. (a) Comparison of polarization curve of $Ru_{0.0328}$-IrO_x, $Ru_{0.0492}$-IrO_x, $Ru_{0.0983}$-IrO_x, and $Ru_{0.148}$-IrO_x. (b) Histogram of overpotentials of $Ru_{0.0328}$-IrO_x, $Ru_{0.0492}$-IrO_x, $Ru_{0.0738}$-IrO_x, $Ru_{0.0983}$-IrO_x, $Ru_{0.148}$-IrO_x, C-RuO_2 and C-IrO_2 at 10 mA cm^{-2} current density. (c) C_{dl} plots for $Ru_{0.0328}$-IrO_x, $Ru_{0.0492}$-IrO_x, $Ru_{0.0983}$-IrO_x, and $Ru_{0.148}$-IrO_x. (d) Tafel plots for $Ru_{0.0328}$-IrO_x, $Ru_{0.0492}$-IrO_x, $Ru_{0.0983}$-IrO_x, and $Ru_{0.148}$-IrO_x. (e) EIS plots for $Ru_{0.0328}$-IrO_x, $Ru_{0.0492}$-IrO_x, $Ru_{0.0983}$-IrO_x, and $Ru_{0.148}$-IrO_x. (f) Chronopotential test plots for $Ru_{0.0328}$-IrO_x, $Ru_{0.0492}$-IrO_x, $Ru_{0.0983}$-IrO_x, and $Ru_{0.148}$-IrO_x.

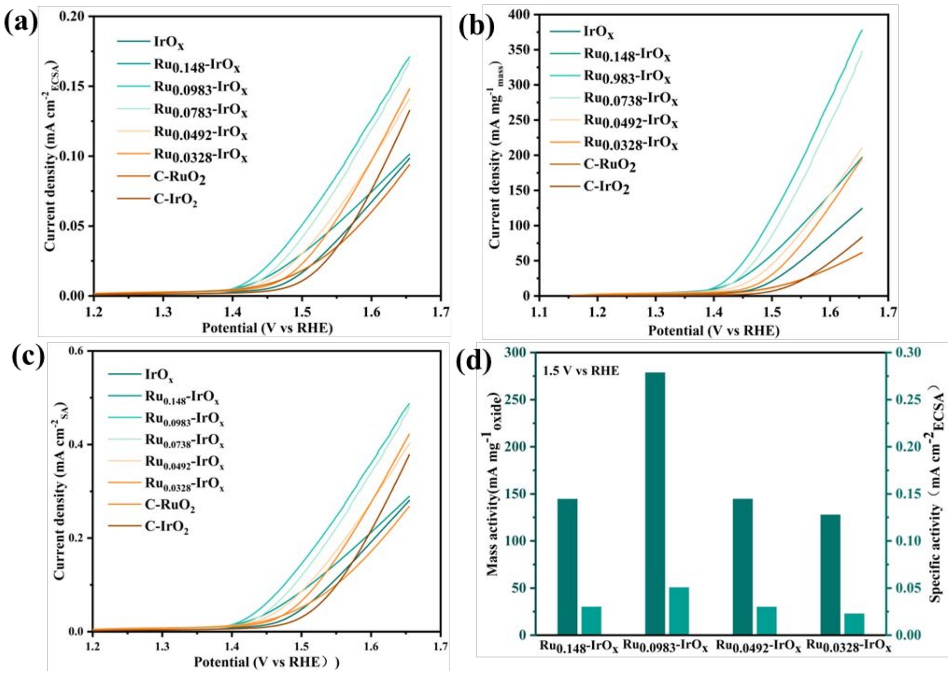

Figure S11. (a) ECSA-normalised polarisation curves for IrO_x, $Ru_{0.0328}$-IrO_x, $Ru_{0.0492}$-IrO_x, $Ru_{0.0738}$-IrO_x, $Ru_{0.0983}$-IrO_x, $Ru_{0.148}$-IrO_x, C-RuO_2, and C-IrO_2. (b) Mass-normalised polarisation curves for IrO_x, $Ru_{0.0328}$-IrO_x, $Ru_{0.0492}$-IrO_x, $Ru_{0.0738}$-IrO_x, $Ru_{0.0983}$-IrO_x, $Ru_{0.148}$-IrO_x, C-RuO_2 and C-IrO_2. (c) SA-normalised polarisation curves for IrO_x, $Ru_{0.0328}$-IrO_x, $Ru_{0.0492}$-IrO_x, $Ru_{0.0738}$-IrO_x, $Ru_{0.0983}$-IrO_x, $Ru_{0.148}$-IrO_x, C-RuO_2 and C-IrO_2. (d) Histogram comparing mass activity (normalized by catalyst loading) and intrinsic activity (normalized by ECSA) at 1.5 V vs. RHE.

Table S4. Comparison of the surface parameters and mass activity for electrocatalysts investigated in 0.5 M H_2SO_4

Sample	C_{dl} (mF cm^{-2})	R_f [a]	Surface area [b] (m^2 g_{oxide}^{-1})	ECSA [c] (mA cm^{-2})	Mass activity [d] (mA mg_{oxide})	Specific activity [e] (mA cm^{-2}$_{ECSA}$)
**IrO_x**	21.6	360	126.3	360	21.11228	0.01671
**$Ru_{0.148}$-IrO_x**	33.24	554	194.4	554	58.80702	0.03025
**$Ru_{0.0983}$-IrO_x**	37.8	630	221.05	630	112.35088	0.05083
**$Ru_{0.0738}$-IrO_x**	35.3	588.3	206.4	588.3	86.07018	0.0417
**$Ru_{0.0492}$-IrO_x**	25.5	425	149.1	425	45.05263	0.03021
**$Ru_{0.0328}$-IrO_x**	22.6	376.7	132.2	376.7	30.34035	0.02295
**C-RuO_2**	5.2	86.7	30.4	86.7	39.36842	0.01817
**C-IrO_2**	4.76	79.3	27.8	79.3	6.77895	0.01078

a: the R_f was calculated by dividing C_{dl} by the capacitance of ideal planar metal oxides with smooth surface, which was taken as 0.06 mF cm^{-2}.

b: the surface area was calculated by multiplying the electrode geometrical area by R_f and then normalized by taking into account the loading mass of electrocatalysts.

c: Multiplying R_f by the catalyst geometric area yields ECSA.

d: Mass activity was obtained from the mass normalized to the current density values at an overpotential of 270 mV.

e: Specific activity was derived from the ECSA normalized to the current density value at an overpotential of 270 mV.

Table S5. The turnover frequency (TOF value) of catalysts at an overpotential of 300 mV.

**Sample**	**TOF value**
**IrO_x**	0.21 S^{-1}
**$Ru_{0.148}$-IrO_x**	0.59 S^{-1}
**$Ru_{0.0983}$-IrO_x**	0.62 S^{-1}
**$Ru_{0.0738}$-IrO_x**	0.67 S^{-1}
**$Ru_{0.0492}$-IrO_x**	0.45 S^{-1}
**$Ru_{0.0328}$-IrO_x**	0.29 S^{-1}
**C-RuO_2**	0.03 S^{-1}
**C-IrO_2**	0.04 S^{-1}

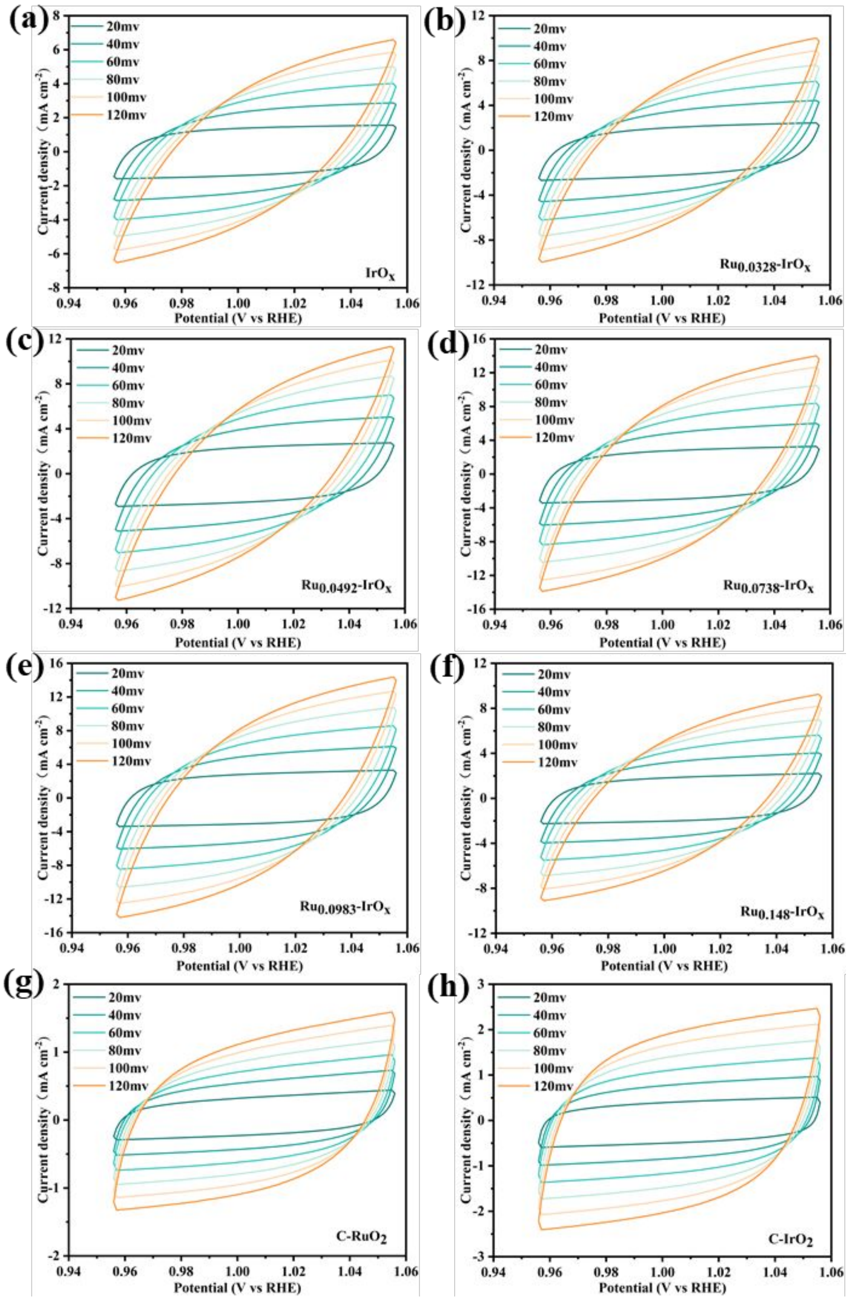

Figure S12. (a-h) Cyclic voltammetric curves for IrO_x, $Ru_{0.0328}$-IrO_x, $Ru_{0.0492}$-IrO_x, $Ru_{0.0738}$-IrO_x, $Ru_{0.0983}$-IrO_x, $Ru_{0.148}$-IrO_x, C-RuO_2 and C-IrO_2 (20mV-120mV).

.

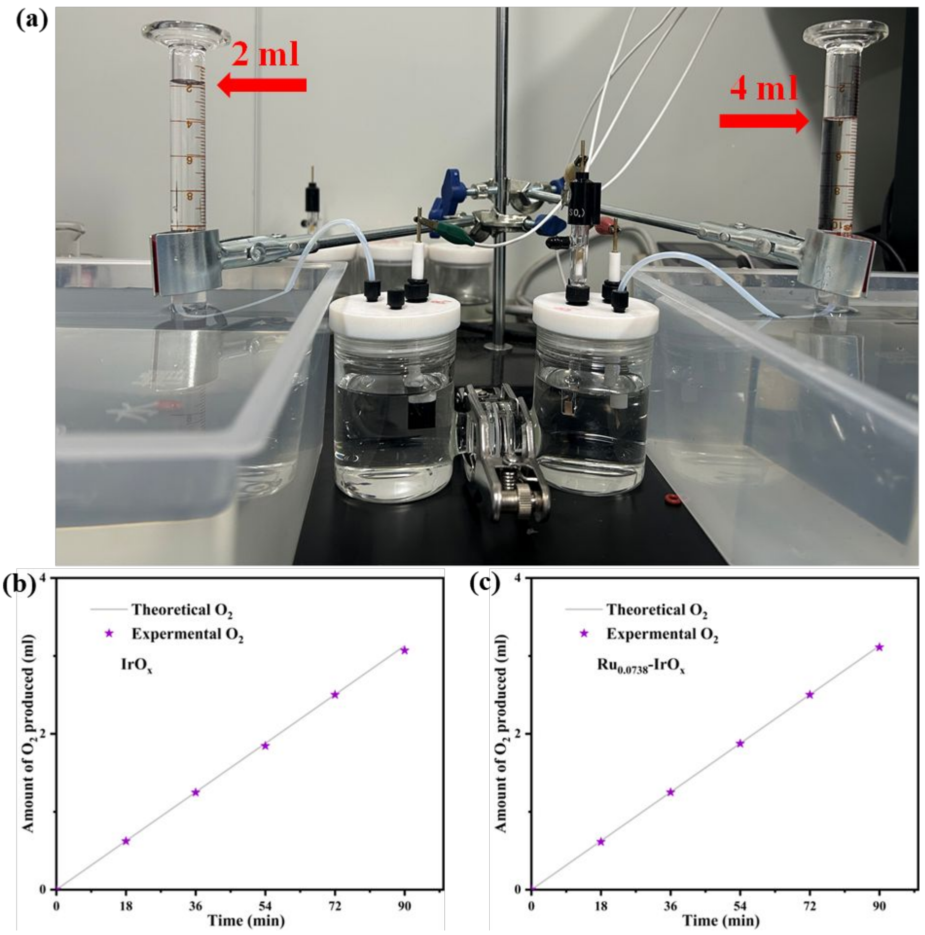

Figure S13. (a) Diagram of the apparatus for testing the amount of oxygen produced by IrO_x and $Ru_{0.0738}$-IrO_x in the OER process by the drainage method. (b) Comparison of theoretically calculated and actual amounts of gas produced by IrO_x in 0.5 M H_2SO_4. (c) Comparison of the theoretically calculated and actual amounts of gas produced by $Ru_{0.0738}$-IrO_x in 0.5 M H_2SO_4.

Table S6. The actual volume of oxygen produced by IrO_x and $Ru_{0.0738}$-IrO_x catalysts in 0.5 M H_2SO_4 and the corresponding FE were tested by the drainage method.

Electrolyte	Samples	Experimental O_2/ml	FE/%
**0.5M H_2SO_4**	**IrO_x**	3.07	98.1
	**$Ru_{0.0738}$-IrO_x**	3.11	99.4

Table S7. Comparison of the overpotentials of $Ru_{0.0738}$-IrO_x NSs with recently reported Ir/Ru-based electrocatalysts at 10 mA cm^{-2} in acidic media.

Catalyst	Electrolyte	η at 10 mA cm^{-2} (mV)	Reference
**$Ru_{0.0738}$-IrO_x NSs**	0.5M H_2SO_4	225	This work
**a-m IrOx NMs**	0.5M H_2SO_4	246	Ref[1]
**$FeCoSn(OH)_6$-300**	0.5M H_2SO_4	266	Ref[2]
**p-L-IrO_2**	0.1 M $HClO_4$	270	Ref[3]
**IrO_x**	0.5M H_2SO_4	255	Ref[4]
**Ir/GF**	0.5M H_2SO_4	290	Ref[5]
**Ir/g-C_3N_4/NG**	0.5M H_2SO_4	287	Ref[6]
**Ir-SA@Fe@NCNT**	0.5M H_2SO_4	250	Ref[7]
**IrNiCu DNF**	0.1 M $HClO_4$	300	Ref[8]
**Ir-based nanocages**	0.1 M $HClO_4$	250	Ref[9]
**DNP-IrNi**	0. 5M H_2SO_4	248	Ref[10]
**IrO_2−blue TiO_2**	0.1 M $HClO_4$	342	Ref[11]
**IrO_2@TiN_{1+x}**	0. 5M H_2SO_4	236	Ref[12]
**H-Ti@IrOx**	0. 5M H_2SO_4	277	Ref[13]
**Ir_3CeO_x/C**	0.1 M $HClO_4$	299	Ref[14]
**IrO_x/F-TiO_2**	0. 5M H_2SO_4	272	Ref[15]
**np-IrCr**	0.1 M $HClO_4$	252	Ref[16]
**Au@$AuIr_2$**	0. 5M H_2SO_4	261	Ref[17]
**Ir_1Ru_4/TiC**	0.1 M $HClO_4$	230	Ref[18]
**$Lu_{1.8}Zn_{0.2}Ir_2O_7$**	0.1 M $HClO_4$	331	Ref[19]
**Ir-VI-ado**	1 M H2SO4	270	Ref[20]
**Ta-RuO_2**	0. 5M H2SO4	226	Ref[21]

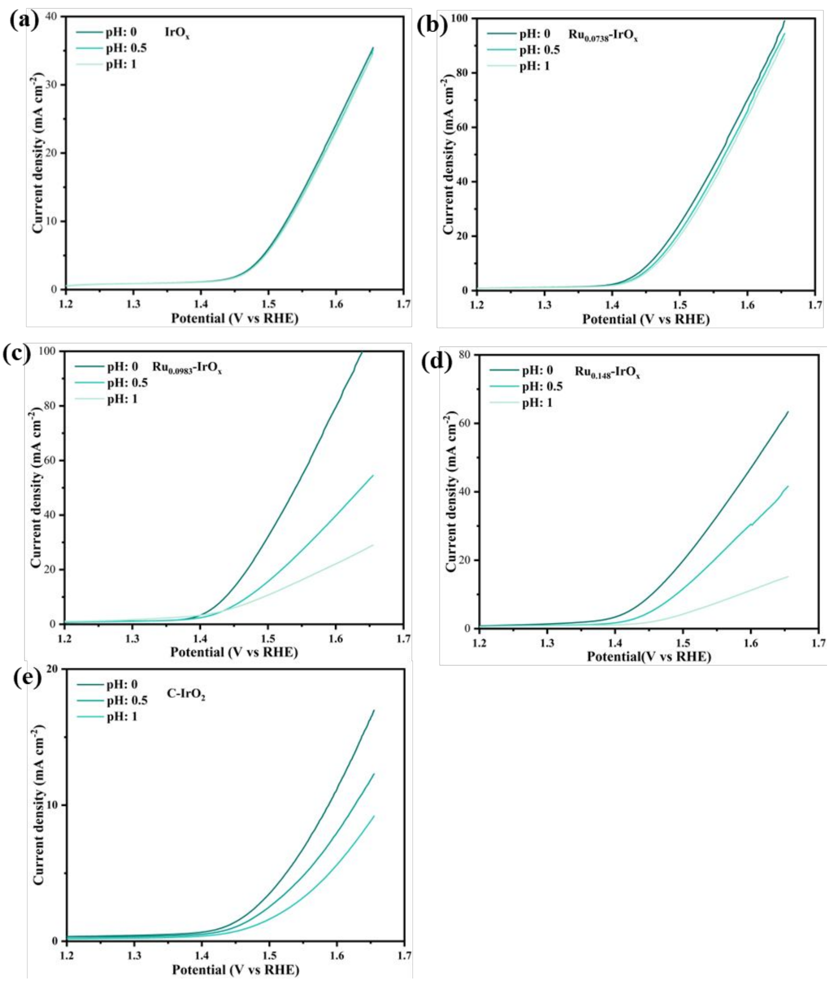

Figure S14. Polarisation curves at different pH values for (a) IrO_x, (b) $Ru_{0.0738}$-IrO_x, (c) $Ru_{0.0983}$-IrO_x, (d) $Ru_{0.148}$-IrO_x, and (e) C-IrO_2.

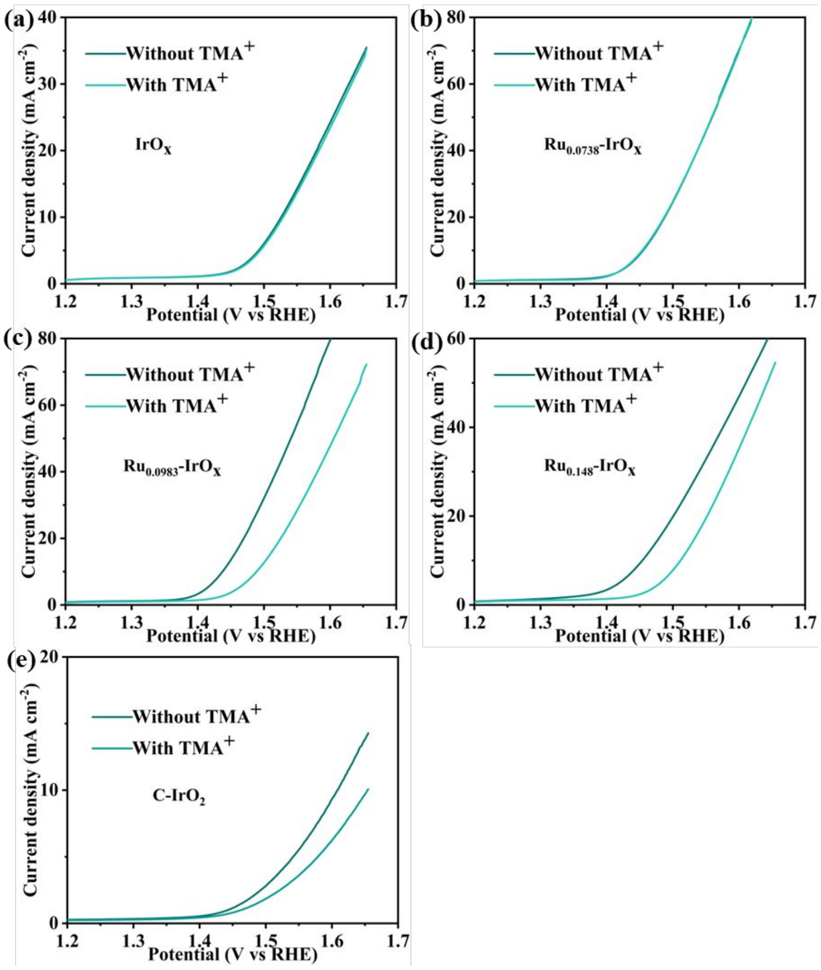

Figure S15. LSV curves of (a) IrO_x, (b) $Ru_{0.0738}$-IrO_x, (c) $Ru_{0.0983}$-IrO_x, (d) $Ru_{0.148}$-IrO_x, and (e) C-IrO_2 with and without addition of TMAOH.

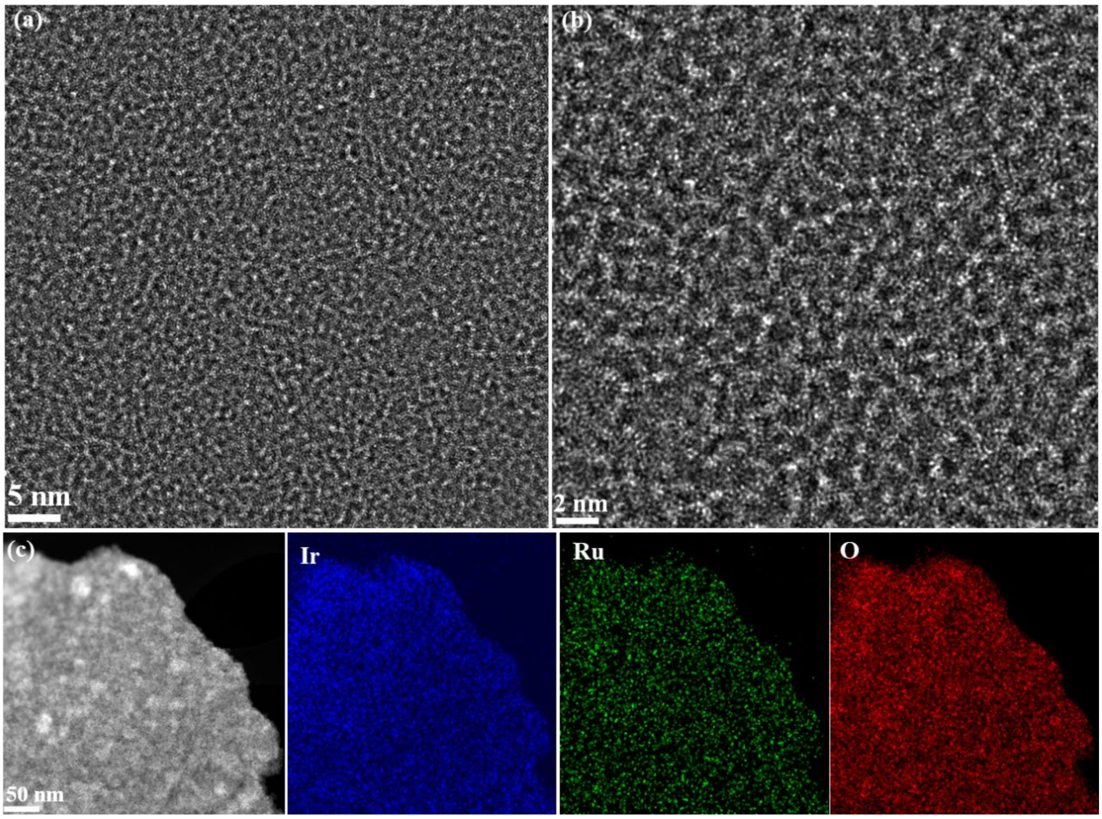

Figure S16. (a) and (b). STEM images of $Ru_{0.0738}$-IrO_x after the stability test. (C) EDS Spectrum of $Ru_{0.0738}$-IrO_x After stability test.

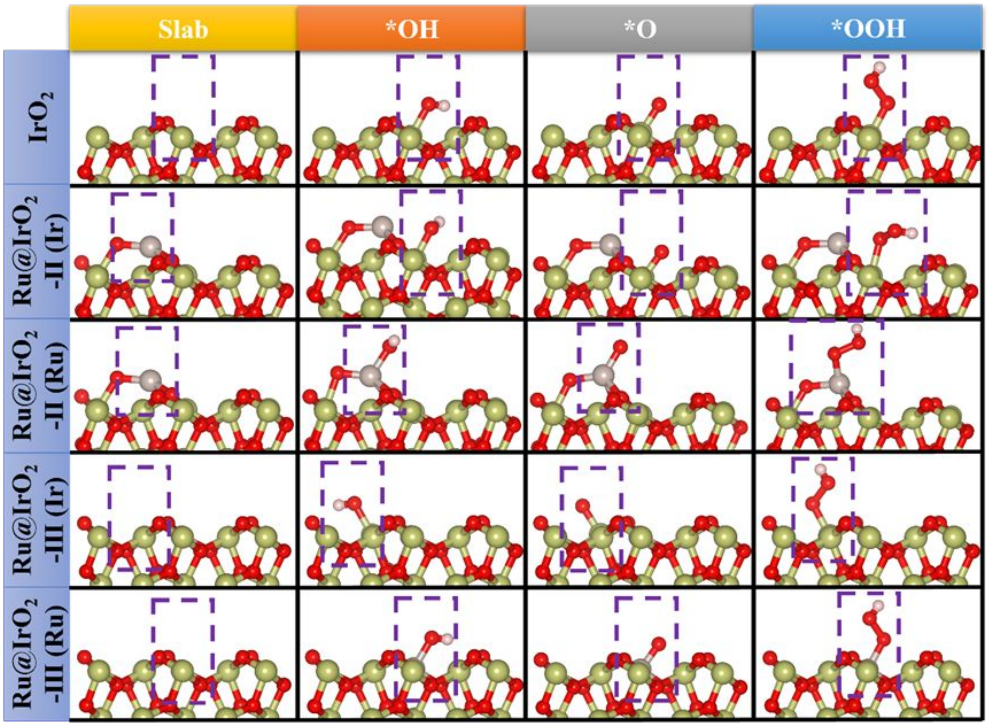

Figure S17. OER reaction process on IrO_x, Ru@IrO_x-II (Ir), Ru@IrO_x-II (Ru), Ru@IrO_x-III (Ir), and Ru@IrO_x-III (Ru) (brackets indicate the corresponding reaction sites and the purple dashed box indicates the intermediate species of the reaction).

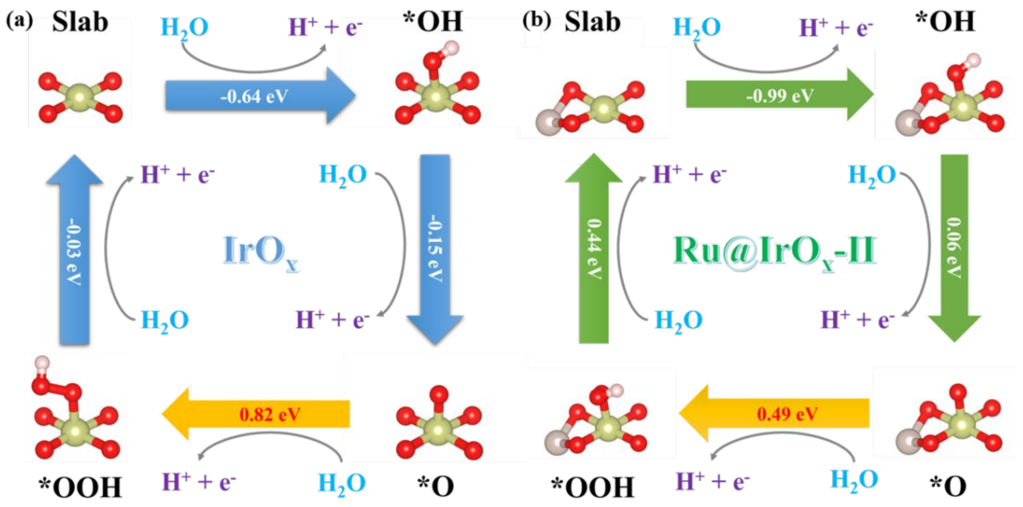

Figure S18. (a) OER reaction paths on the surface of IrO_x (Ir) and (b) Ru@IrO_x-II (Ir) (brackets denote the reacted sites, orange arrows denote the RDS).

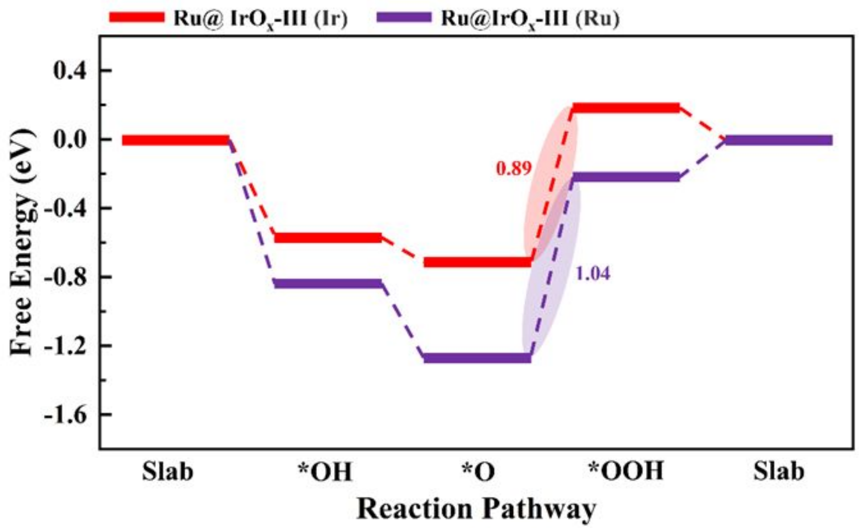

Figure S19. OER free energy diagram of the Ir, Ru sites on Ru@IrO$_x$-III (reaction mechanisms in brackets, shaded positions indicate RDS)

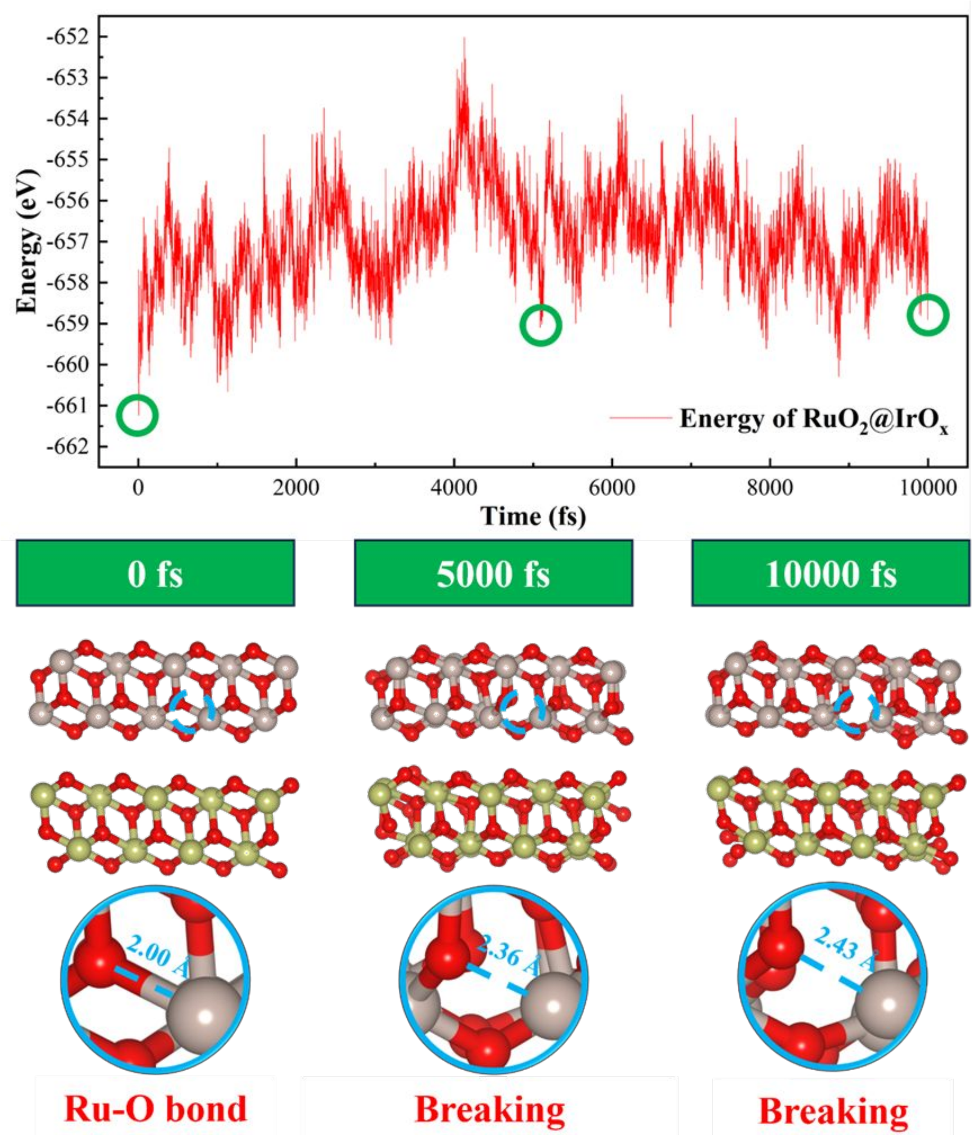

Figure S20. Molecular dynamics (AIMD) simulation of RuO_2@IrO_x at 500 K and structural modeling at 0 fs, 5000 fs, and 10000 fs during the simulation.

Reference

(1) Li, Y.; Yu, G.; Li, J.; Bian, Z.; Han, X.; Wu, B.; Wu, G.; Yang, Q.; Hong, X. Universal Synthesis of Amorphous Metal Oxide Nanomeshes. Small 2024, 20 (33), 2401162, DOI: 10.1002/smll.202401162.

(2) Liu, S.; Geng, S.; Li, L.; Zhang, Y.; Ren, G.; Huang, B.; Hu, Z.; Lee, J.F.; Lai, Y.H.; Chu, Y.H.; et al. A top-down strategy for amorphization of hydroxyl compounds for electrocatalytic oxygen evolution. Nature Communications 2022, 13 (1), 1187 (2022), DOI: 10.1038/s41467-022-28888-3.

(3) Xie, Z.; Liang, X.; Kang, Z.; Zou, Y.; Wang, X.; Wu, Y. A.; King, G.; Liu, Q.; Huang, Y.; Zhao, X.; et al. High-Porosity, Layered Iridium Oxide as an Efficient, Durable Anode Catalyst for Water Splitting. Ccs Chemistry 2025, 7 (1), 216-228, DOI: 10.31635/ccschem.024.202303586.

(4) Yu, G.; Li, R.; Hu, Y.; Lin, X.; Lin, Z.; Wu, D.; Wang, G.; Hong, X. Supporting IrO_x nanosheets on hollow TiO_2 for highly efficient acidic water splitting. Nano Research 2024, 17 (8), 6903-6909, Article. DOI: 10.1007/s12274-024-6681-7.

(5) Zhang, J.; Wang, G.; Liao, Z.; Zhang, P.; Wang, F.; Zhuang, X.; Zschech, E.; Feng, X. Iridium nanoparticles anchored on 3D graphite foam as a bifunctional electrocatalyst for excellent overall water splitting in acidic solution. Nano Energy 2017, 40, 27-33, DOI: 10.1016/j.nanoen.2017.07.054.

(6) Jiang, B.; Wang, T.; Cheng, Y.; Liao, F.; Wu, K.; Shao, M. Ir/g-C_3N_4/Nitrogen-Doped Graphene Nanocomposites as Bifunctional Electrocatalysts for Overall Water Splitting in Acidic Electrolytes. Acs Applied Materials & Interfaces 2018, 10 (45), 39161-39167, DOI: 10.1021/acsami.8b11970.

(7) Luo, F.; Hu, H.; Zhao, X.; Yang, Z.; Zhang, Q.; Xu, J.; Kaneko, T.; Yoshida, Y.; Zhu, C.; Cai, W. Robust and Stable Acidic Overall Water Splitting on Ir Single Atoms. Nano Letters 2020, 20 (3), 2120-2128, DOI: 10.1021/acs.nanolett.0c00127.

(8) Park, J.; Sa, Y. J.; Baik, H.; Kwon, T.; Joo, S. H.; Lee, K. Iridium-Based Multimetallic Nanoframe@Nanoframe Structure: An Efficient and Robust Electrocatalyst toward Oxygen Evolution Reaction. Acs Nano 2017, 11 (6), 5500-5509, DOI: 10.1021/acsnano.7b00233.

(9) Zhu, J.; Chen, Z.; Xie, M.; Lyu, Z.; Chi, M.; Mavrikakis, M.; Jin, W.; Xia, Y. Iridium-Based Cubic Nanocages with 1.1-nm-Thick Walls: A Highly Efficient and Durable Electrocatalyst for Water Oxidation in an Acidic Medium. Angewandte Chemie-International Edition 2019, 58 (22), 7244-7248, DOI: 10.1002/anie.201901732.

(10) Yeo, K.R.; Lee, K.S.; Kim, H.; Lee, J.; Kim, S.K. A highly active and stable 3D dandelion spore-structured self-supporting Ir-based electrocatalyst for proton exchange membrane water electrolysis fabricated using structural reconstruction. Energy & Environmental Science 2022, 15 (8), 3449-3461, DOI: 10.1039/d2ee01042a.

(11) Nguyen, C. T. K.; Ngoc Quang, T.; Thi Anh, L.; Lee, H. Covalently Bonded Ir(IV) on Conducted Blue TiO_2 for Efficient Electrocatalytic Oxygen Evolution Reaction in Acid Media. Catalysts 2021, 11 (10), 1176, DOI: 10.3390/catal11101176.

(12) Wang, S.; Lv, H.; Bi, S.; Li, T.; Sun, Y.; Ji, W.; Feng, C.; Zhang, C. Defects tailoring IrO_2@TiN_{1+x} nano-heterojunctions for superior water oxidation activity and stability. Materials Chemistry Frontiers 2021, 5 (22), 8047-8055, DOI: 10.1039/d1qm00845e.

(13) Yu, Z.; Xu, J.; Li, Y.; Wei, B.; Zhang, N.; Li, Y.; Bondarchuk, O.; Miao, H.; Araujo, A.; Wang, Z.; et al. Ultrafine oxygen-defective iridium oxide nanoclusters for efficient and durable water oxidation at high current densities in acidic media. Journal of Materials Chemistry A 2020, 8 (46), 24743-24751, DOI: 10.1039/d0ta07093a.

(14) Zhao, X.; Chang, Y.; He, X.; Zhang, H.; Jia, J.; Jia, M. Understanding ultra-dispersed CeO_x modified iridium clusters as bifunction electrocatalyst for high-efficiency water splitting in acid electrolytes. Journal of Rare Earths 2023, 41 (2), 208-214, DOI: 10.1016/j.jre.2022.01.013.

(15) Li, G.; Xu, X.; Liu, H.; Yang, X.; Lin, M.C. Enhanced Electrocatalytic Performance of IrO_x by Employing F-Doped TiO_2 as Support towards Acidic Oxygen Evolution Reaction. Chemcatchem 2022, 14, e202201039, DOI: 10.1002/cctc.202201039.

(16) Chatterjee, S.; Intikhab, S.; Profitt, L.; Li, Y.; Natu, V.; Gawas, R.; Snyder, J. Nanoporous multimetallic Ir alloys as efficient and stable electrocatalysts for acidic

oxygen evolution reactions. Journal of Catalysis 2021, 393, 303-312, DOI: 10.1016/j.jcat.2020.11.038.

(17) Wang, H.; Chen, Z.N.; Wu, D.; Cao, M.; Sun, F.; Zhang, H.; You, H.; Zhuang, W.; Cao, R. Significantly Enhanced Overall Water Splitting Performance by Partial Oxidation of Ir through Au Modification in Core-Shell Alloy Structure. Journal of the American Chemical Society 2021, 143 (12), 4639-4645, DOI: 10.1021/jacs.0c12740.

(18) Zhang, J.; Cao, X.; Jiang, Y.F.; Hung, S.F.; Liu, W.; Yang, H. B.; Xu, C.Q.; Li, D.S.; Zhang, T.; Li, Y.; et al. Surface enrichment of Ir on the IrRu alloy for efficient and stable water oxidation catalysis in acid. Chemical Science 2022, 13 (41), 12114-12121, DOI: 10.1039/d2sc03947h.

(19) Zhu, L.; Ma, C.; Wang, Z.; Gong, X.; Cao, L.; Yang, J. Tuning the hybridization state of Ir-O to improve the OER activity and stability of iridium pyrochlore via Zn doping. Applied Surface Science 2022, 576, 151840, DOI: 10.1016/j.apsusc.2021.151840.

(20) Li, A.; Kong, S.; Adachi, K.; Ooka, H.; Fushimi, K.; Jiang, Q.; Ofuchi, H.; Hamamoto, S.; Oura, M.; Higashi, K.; et al. Atomically dispersed hexavalent iridium oxide from MnO_2 reduction for oxygen evolution catalysis. Science 2024, 384 (6696), 666-670, DOI: 10.1126/science.adg5193.

(21) Zhang, J. H.; Fu, X. B.; Kwon, S.; Chen, K. F.; Liu, X. Z.; Yang, J.; Sun, H. R.; Wang, Y. C.; Uchiyama, T.; Uchimoto, Y.; et al. Tantalum-stabilized ruthenium oxide

electrocatalysts for industrial water electrolysis. Science 2025, 387 (6729), 48-55, DOI: 10.1126/science.ado9938.
